# Effects of different frequencies of repetitive transcranial magnetic stimulation on sleep disorders and depression in patients with Parkinson’s disease: a systematic review and network meta-analysis

**DOI:** 10.3389/fnagi.2025.1623917

**Published:** 2025-09-08

**Authors:** Yuan Xia, Haili Wan, Xin Hu, Wenhui Sun, Yongjie Li

**Affiliations:** ^1^Department of Rehabilitation and Treatment, Hubei Rehabilitation Hospital, Wuhan, China; ^2^School of Public Finance and Taxation, Central University of Finance and Economics, Beijing, China; ^3^Department of Rehabilitation Medicine, Beijing Jishuitan Hospital Guizhou Hospital, Guizhou Provincial Orthopedics Hospital, Guiyang, China

**Keywords:** Parkinson, sleep disorder, depression, repetitive transcranial magnetic stimulation, meta-analysis

## Abstract

**Background:**

Repetitive transcranial magnetic stimulation (rTMS) has emerged as a promising neuromodulatory approach for alleviating sleep disturbances and depressive symptoms in Parkinson’s disease (PD), yet direct comparisons of different stimulation frequencies remain scarce.

**Objective:**

To evaluate and rank the efficacy of three rTMS frequencies (1 Hz, 5 Hz, and 10 Hz), each combined with conventional therapy, on sleep disorders and depression in PD patients, thereby informing clinical decision-making.

**Methods:**

We conducted a systematic search for randomized controlled trials (RCTs) in PubMed, Embase, the Cochrane Library, Web of Science, ProQuest, China National Knowledge Infrastructure, Wanfang, and the Chinese Scientific and Journal Database. A network meta-analysis was performed to compare the effects of different frequencies of rTMS (1 Hz, 5 Hz, and 10 Hz) on sleep disorders and depression in PD patients.

**Results:**

Thirty-one RCTs involving 1,977 PD patients met inclusion criteria. Compared with conventional treatment alone, adjunctive 5 Hz and 10 Hz rTMS produced significant improvements in both Pittsburgh Sleep Quality Index (PSQI) and Parkinson’s Disease Sleep Scale (PDSS). Although 1 Hz rTMS yielded numerically greater PSQI and PDSS improvements than conventional therapy, these differences did not reach statistical significance, nor did differences between the three stimulation frequencies. In terms of depressive symptoms, all three frequencies (1 Hz, 5 Hz, and 10 Hz) significantly reduced HAMD scores versus standard care, with head-to-head comparisons indicating superior efficacy of 10 Hz over 1 Hz and 5 Hz. The Surface Under the Cumulative Ranking area (SUCRA) consistently identified 10 Hz rTMS as the most effective frequency for PSQI, PDSS, and HAMD outcomes.

**Conclusion:**

Adjunctive rTMS at 1 Hz, 5 Hz, and 10 Hz each confer benefits for sleep and mood in PD patients, but 10 Hz stimulation appears to offer the greatest overall improvement. These findings support the preferential use of 10 Hz rTMS when targeting non-motor symptoms in Parkinson’s disease.

**Systematic review registration:**

https://www.crd.york.ac.uk/PROSPERO/recorddashboard, identifier CRD42024614337.

## 1 Introduction

Parkinson’s disease (PD), which primarily affects middle-aged and older adults, is the world’s second most common neurodegenerative disorder (Rocca, 2018; GBD 2016 Neurology Collaborators., 2019). In addition to its hallmark motor symptoms such as tremor, rigidity and bradykinesia, PD is also characterized by a wide range of non-motor impairments, including mood disturbances, cognitive decline and sleep disorders (Hendricks and Khasawneh, 2021; Kirmani et al., 2021). The exact pathogenesis of PD remains unclear; current evidence suggests that non-motor symptoms predominantly arise from diminished dopaminergic transmission within the midbrain–limbic and midbrain–cortical systems (Moore et al., 2008). Most individuals with PD experience sleep problems early in the disease course or even before overt motor signs appear (Barone et al., 2009). Common sleep disorders in PD include rapid eye movement sleep behavior disorder (RBD), insomnia, restless legs syndrome, sleep-related breathing disturbances and excessive daytime sleepiness (Chahine et al., 2017). These disturbances may result from side effects of dopaminergic medications, neurodegenerative changes in brain structures that regulate sleep and nocturnal motor symptoms (French and Muthusamy, 2016). The regulation of sleep relies on the comprehensive function of multiple brain regions and various neurotransmitters, including dopamine, serotonin, norepinephrine, and other PD-related neurotransmitters (Stefani and Högl, 2020). These neurotransmitters not only regulate sleep disorders but may also be associated with cognitive dysfunction in PD (Yeung and Cavanna, 2014; Maggi et al., 2021; Malhotra, 2022). Emotional disorders involve depression, anxiety, etc. Recent studies have shown that there is a strong correlation between sleep quality and depressive and anxious emotions in PD patients, and the severity of sleep disorders is related to the degree of depression (Kay et al., 2018; Rana et al., 2018). Depression is another prevalent non-motor feature of PD and often has an insidious onset that precedes typical motor manifestations (Langston, 2006; Simonetta-Moreau, 2014). In patients with PD, depressive episodes frequently co-occur with anxiety, irritability, sadness and pessimism about the future, all of which can worsen sleep disturbances (van der Hoek et al., 2011). Depression and sleep disorders frequently co-occur in PD, each exacerbating the other in a self-perpetuating cycle. The close link between sleep dysfunction and depression leads to loss of mobility, reduced functional independence and severe impairments in mood and daily quality of life (Hariz and Forsgren, 2011; Zhao et al., 2021). These challenges also place a heavy burden on families and healthcare systems, since the global economic cost of PD reached an estimated $52 billion in 2017 and is projected to exceed $80 billion by 2040 as populations continue to age (Dorsey et al., 2018). Current management of PD relies mainly on dopaminergic pharmacotherapy, but long-term use of these agents carries risks of adverse effects and may even accelerate neurodegeneration (Jiménez-Urbieta et al., 2015; Seppi et al., 2019). It is therefore critical to explore non-pharmacological interventions that carry fewer risks and can help alleviate both sleep disturbances and depression in PD patients.

Repetitive transcranial magnetic stimulation (rTMS) is a non-invasive neuromodulation technique grounded in electromagnetic induction; a pulsed magnetic field is applied to the skull surface to induce weak electrical currents in targeted brain regions (Chail et al., 2018). Depending on the frequency of the stimulation pulses, rTMS is classified as either low-frequency (≤1 Hz, low frequency rTMS, LF-rTMS) or high-frequency (>1 Hz, high frequency rTMS, HF-rTMS) stimulation (Xia et al., 2022). Previous studies have demonstrated that its therapeutic effects arise from bidirectional modulation of cortical excitability: low-frequency rTMS reduces excitability, while high-frequency rTMS enhances it (Cirillo et al., 2017). However, direct comparisons across frequencies are scarce, so this study employed a network meta-analysis (NMA) to evaluate how different rTMS frequencies affect sleep disorders and depression in PD patients and to identify the optimal stimulation frequency for clinical use.

## 2 Materials and methods

This systematic review was conducted in accordance with Preferred Reporting Items for Systematic Evaluation and Meta-Analysis statement (PRISMA) guidelines (Hutton et al., 2015) and the Cochrane Handbook for Systematic Reviews of Interventions to ensure methodological rigor. The protocol was registered with PROSPERO under registration number CRD42024614337.

### 2.1 Search strategy

Systematic searches were conducted on PubMed, EMBASE, Cochrane Library, Web of Science, ProQuest, China National Knowledge Infrastructure, Wanfang Database and Chinese Scientific and Journal Database (VIP) before February 2025 for randomized controlled trials (RCTs) on the effects of rTMS stimulation on sleep disorders and depression in PD patients. Using a combination of logical connective, medical MeSH, and free-text terms, search terms included: “Parkinson’s Disease,” “Parkinson,” “Parkinson’s,” “Parkinsonism,” “Repetitive Transcranial Magnetic Stimulation,” “Transcranial Magnetic Stimulation,” “rTMS,” “Sleep,” “Sleep disorders,” “Depression.” See Supplementary Table 1 for detailed search strategies for PubMed.

### 2.2 Inclusion criteria

Inclusion criteria were defined according to the PICOS framework (Population, Interventions, Comparators, Outcomes, and Study Design).

1. Population: Participants diagnosed with PD who reported sleep disorders, were over 18 years of age, of any gender, and provided informed consent.
2. Interventions: Experimental groups received low-frequency rTMS (LF-rTMS) or high-frequency rTMS (HF-rTMS); all interventions were given in addition to standard care, which encompassed conventional antiparkinsonian medications or routine rehabilitation.
3. Comparators: Different frequencies of rTMS, no stimulation, or sham stimulation (the latter referring to the absence of effective magnetic stimulation with only the sound simulated); all comparators were given in addition to standard care, which encompassed conventional antiparkinsonian medications or routine rehabilitation.
4. Outcomes: Primary outcomes included the Pittsburgh Sleep Quality Index (PSQI), Parkinson’s Disease Sleep Scale (PDSS), and Hamilton Rating Scale for Depression (HAMD); secondary outcomes comprised adverse events.
5. Study design: Only randomized controlled trials (RCTs) in human participants were eligible.

### 2.3 Exclusion criteria

1. Sleep disorders were not attributable to PD; (2) data on any primary or secondary outcomes were unavailable; (3) the full text could not be retrieved; (4) the study was a duplicate publication.

### 2.4 Study selection

All retrieved records were first imported into EndNoteX9 for duplicate removal. Two independent reviewers then screened titles and abstracts to exclude studies that did not meet the inclusion criteria. The full text of all remaining articles was read and assessed for eligibility, and any disagreements were resolved by a third reviewer (S.W.H).

### 2.5 Data extraction

Data extraction was performed independently by two investigators (X.Y and L.Y.J). For each included study, we recorded the first author, year and country of publication, sample size, participant age, intervention type, stimulation parameters, stimulation site and outcome measures. All data were entered into an Excel spreadsheet and cross-checked by both investigators; any discrepancies were adjudicated by a third investigator (S.W.H). When multiple reports used the same data set, we selected the publication with the higher quality score or, if scores were equal, the larger sample size.

### 2.6 Quality assessment

The quality of the included studies was assessed by two independent reviewers (W.H.L and H.X) using the Cochrane Risk of Bias Tool 2.0 (RoB 2.0) (Sterne et al., 2019) and the Physiotherapy Evidence Database (PEDro) scale. The evaluation of RoB 2.0 encompasses the randomization process, deviations from intended interventions, missing outcome data, measurement of outcomes, and selection of reported results. The risk of bias in each domain can be categorized into three levels: “low risk,” “some concerns,” and “high risk.” If the assessment results in all domains are “low risk,” then the overall risk of bias is considered “low risk”; if some domains have “some concerns” and none have “high risk,” then the overall risk of bias is “some concerns”; if even one domain is rated “high risk,” the overall risk of bias is considered “high risk.” The PEDro scale consists of 11 items, with the first item not contributing to the total score, which totals 10 points. Studies with a score of ≥6 (6/10) are considered “good” quality, 4–5 are “fair” quality, and <4 are “poor” quality. Any disagreements in the assessment process were decided by a third investigator (S.W.H).

### 2.7 Statistical analysis

Network meta-analysis was conducted in Stata 16.0. Because all outcomes were continuous variables measured on the same scale, we used weighted mean differences (WMD) and 95 percent confidence intervals as effect sizes. We visualized comparisons in a network evidence diagram in which each node represents an intervention (node size proportional to total sample size) and each connecting line represents a direct comparison (line thickness proportional to number of studies). Consistency between direct and indirect evidence was assessed via ring inconsistency testing; a 95% CI for the inconsistency factor that included zero indicated good agreement. Pairwise comparative forest plots were generated to display intervention effects; effect sizes lying on one side of the null line with confidence intervals that did not cross 0 were considered statistically significant. We calculated Surface Under the Cumulative Ranking area (SUCRA) to rank interventions. The SUCRA value ranges from 0 to 100, where higher values indicate superior intervention efficacy and lower values correspond to diminished effectiveness. Finally, used funnel plots to evaluate publication bias and other small-study effects. Subgroup and sensitivity analyses were conducted to assess the robustness of our findings. Studies were stratified into two subgroups based on total pulse count (≤600 pulses and >600 pulses); Sensitivity analyses were conducted after excluding studies with sample sizes <10, PEDro scores < 6, and Hoehn & Yahr stages (H&Y) > 3. Publication bias and small-study effects were evaluated using funnel plots in Stata 16.0.

## 3 Results

### 3.1 Study selection

The initial search yielded 5303 records. After removing 1332 duplicates, 3 971 records remained. Title and abstract screening excluded 3862 records, leaving 109 articles for full-text review; 78 of these were excluded and 31 trials were included in the network meta-analysis.

**FIGURE 1:**
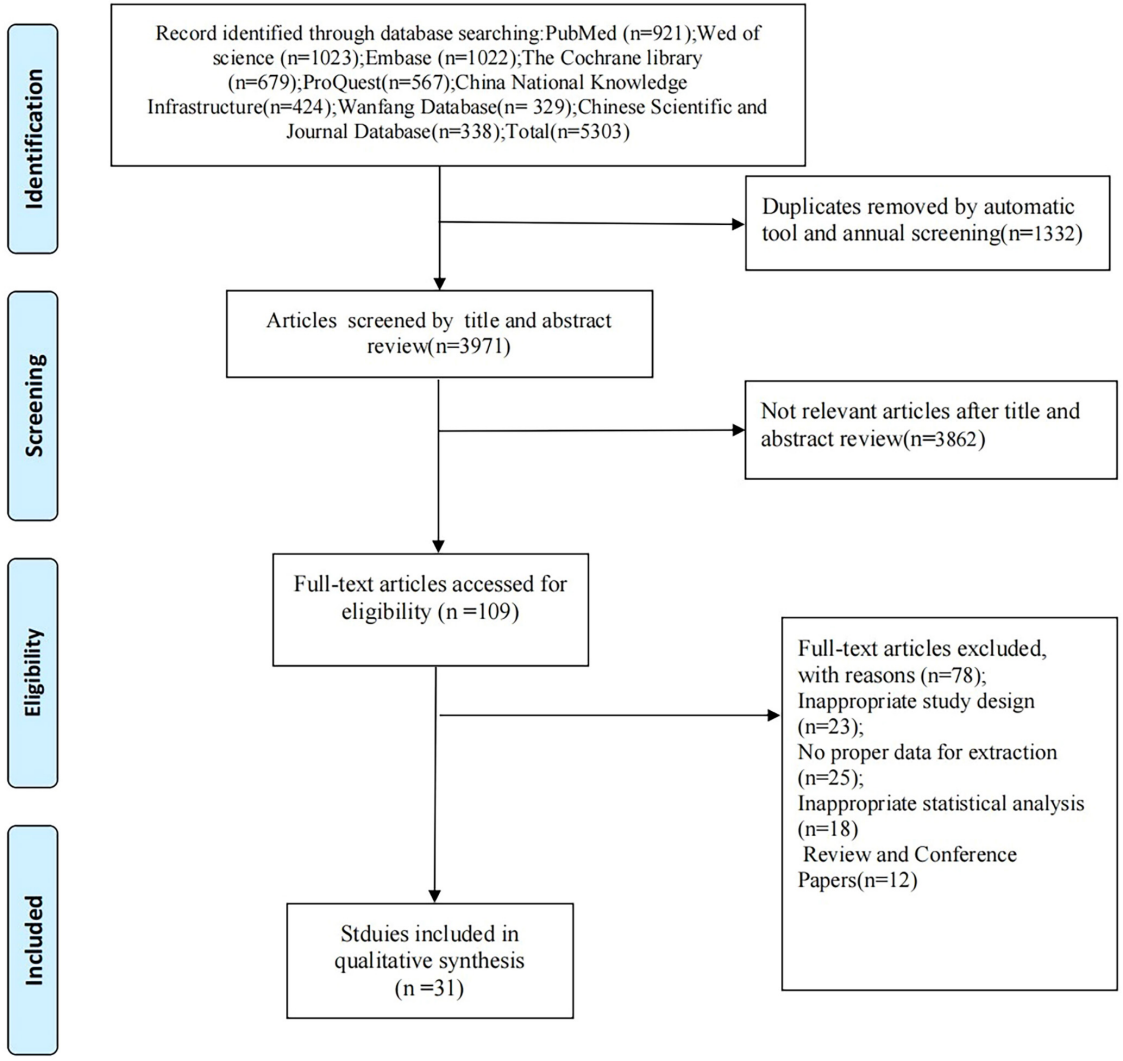
Flow diagram of the eligible studies selection process.

Figure 1 depicts the screening and selection process of the articles.

### 3.2 Characteristics of the included studies

In total, 1977 Parkinson’s disease patients were enrolled. Twenty-four trials were published in Chinese and seven in English, with publication dates ranging from 2013 to 2024. In the control arms, one trial combined rehabilitation training with sham stimulation and the remainder combined medication with sham stimulation. In the experimental arms, 12 trials applied low-frequency rTMS at 1 Hz; 10 trials applied high-frequency rTMS at 5 Hz; and 12 trials applied high-frequency rTMS at 10 Hz. 14 studies reported PSQI, 8 studies reported PDSS only, and 19 studies reported HAMD. Detailed characteristics of the included studies are shown in Table 1. (Moore et al., 2008; Zhuohua et al., 2013; Wenjing et al., 2014; Brys et al., 2016; Shin et al., 2016; Fengju et al., 2017; Yu Wen-wen and Hai-rong, 2017; Ding and Xu, 2018; Hua et al., 2019; Chao, 2020; Lai Jinghui et al., 2020; Zhuang et al., 2020; Chinese Journal of Practical Nervous Diseases, 2021; Dai Wei-zheng et al., 2021; Jia-Jin, 2021; Wang Dong and Yuanyu, 2021; Wang Yajun, 2021; Chen et al., 2022; Ouyang Gui-lan, 2022; Qingping et al., 2022; Yu Xiaolan, 2022; Zhang and Sha, 2022; Hu Xiaoying et al., 2023; Jiang et al., 2023; Li, 2023; Shaheen et al., 2023; Xue et al., 2023; Zhao Rong, 2023; Lei Meng and Jichao, 2024; Qin Xi-xiang et al., 2024; Wu et al., 2024; Zhang et al., 2025).

**TABLE 1:** Characteristics of the included studies.

References	Country	Sample size (E/C)	Mean age (E/C, year)	Duration of illness (E/C, year)	Interventions (E/C, year)	rTMS target	rTMS frequency (Hz)	No. of pulses	Outcomes
Chinese Journal of Practical Nervous Diseases, 2021	China	34/42	62.06 ± 9.02/65.19 ± 10.34	6.09 ± 3.32/7.71 ± 5.57	HF-rTMS + CT/CT + Sham	Left DLPFC	10 Hz	1200	①
Ding and Xu, 2018	China	42/42	66.30 ± 7.50/67.60 ± 8.60	3.24 ± 1.23/3.16 ± 1.42	HF-rTMS + CT/CT + Sham	Bilateral dorsolateral frontal lobes	5 Hz	–	① ②
Hu Xiaoying et al., 2023	China	47/47	67.93 ± 5.39/68.13 ± 5.41	4.30 ± 0.71/4.21 ± 0.69	HF-rTMS + CT/CT + Sham	Bilateral dorsolateral prefrontal cortex	5 Hz	600	① ②
Lai Jinghui et al., 2020	China	20/20	69.55 ± 1.64/71.20 ± 1.67	4.23 ± 0.61/5.50 ± 1.28	HF-rTMS + CT/CT + Sham	SMA	10 Hz	1200	②
Lei Meng and Jichao, 2024	China	48/48	61.48 ± 4.82/60.91 ± 5.33	5.37 ± 1.09/5.63 ± 1.18	HF-rTMS + CT/CT + Sham	F3	10 Hz	2400	①
Xue et al., 2023	China	51/51	64.17 ± 5.42/64.02 ± 5.67	6.17 ± 2.24/6.12 ± 2.13	HF-rTMS + CT/CT + Sham	Left DLPFC	5 Hz	600	① ③
Ouyang Gui-lan, 2022	China	48/48	61.00 ± 9.00/60.00 ± 8. 80	6.10 ± 4.40/5.50 ± 4.50	LF-rTMS + CT/CT + Sham	M1	1 Hz	2800	①
Qin Xi-xiang et al., 2024	China	46/46	63.90 ± 7.20/64.50 ± 6.70	7.70 ± 1.90/7.40 ± 1.70	HF-rTMS + CT/CT + Sham	Bilateral dorsolateral prefrontal cortex	5 Hz	1600	② ③
Wang Yajun, 2021	China	25/25	67.55 ± 6.89/68.00 ± 5.65	4.56 ± 2.92/3.00 ± 1.92	LF-rTMS + CT/CT + Sham	Dorsolateral prefrontal cortex and occipital region	1 Hz	600	①
Jia-Jin, 2021	China	10/10	63.90 ± 8.66/65.20 ± 4.24	6.35 ± 3.64/5.60 ± 3.02	LF-rTMS + CT/CT + Sham	Right DLPFC	1 Hz	1200	① ③
Zhuohua et al., 2013	China	29/29	62.12 ± 7.51/63.94 ± 7.39	6.75 ± 3.12/6.68 ± 3.28	LF-rTMS + CT/CT + Sham	Right frontal lobe	1 Hz	–	①
Yu Wen-wen and Hai-rong, 2017	China	31/33	67.25 ± 6.71/68.00 ± 7.56	2.76 ± 1.56/2.64 ± 1.49	HF-rTMS + CT/CT + Sham	Left DLPFC	5 Hz	1600	② ③
Fengju et al., 2017	China	34/33	62.37 ± 7.90/63.50 ± 6.40	6.10 ± 1.70/6.30 ± 1.40	HF-rTMS + CT/CT + Sham	Lateral left frontal lobe	10 Hz	800	① ③
Zhao Rong, 2023	China	32/32/32	62.30 ± 10.51/60.39 ± 8.64/ 62.10 ± 10.62	–	HF-rTMS + CT/LF-rTMS + CT/CT	Bilateral M1 region and cerebellum	10 Hz/1 Hz	1750/1050	②
Wu et al., 2024	China	34/29	63.00 ± 11.33/65.00 ± 7.03	5.0 ± 3.70/5.0 ± 4.59	LF-rTMS + CT/CT + Sham	Right DLPFC	1 Hz	1200	①
Zhuang et al., 2020	China	19/14	60.58 ± 9.21/61.57 ± 13.25	5.86 ± 4.35/5.71 ± 3.77	LF-rTMS + CT/CT + Sham	Right DLPFC	1 Hz	1200	① ③
Li, 2023	China	15/15	58.94 ± 2.14/58.28 ± 2.31	–	HF-rTMS + CT/LF-rTMS + CT	Right DLPFC	10 Hz/1 Hz	1200	① ②
Shaheen et al., 2023	Egypt	20/20	61.60 ± 7.30/61.10 ± 6.30	3.60 ± 2.30/3.40 ± 2.20	HF-rTMS + CT/CT + Sham	Bilateral parietal cortex	10 Hz	1000	①
Zhang et al., 2025	China	38/40	65.38 ± 8.34/63.90 ± 7.75	4.40 ± 1.45/4.25 ± 1.69	HF-rTMS + CT/CT + Sham	Left DLPFC	5 Hz	1600	② ③
Dai Wei-zheng et al., 2021	China	40/40	60.78 ± 7.02/61.10 ± 6.44	2.34 ± 0.87/2.25 ± 0.66	HF-rTMS + CT/CT	Left DLPFC	5 Hz	1600	③
Wang Dong and Yuanyu, 2021	China	44/44	60.60 ± 9.50/60.20 ± 10.12	1.57 ± 0.34/1.45 ± 0.31	HF-rTMS + CT/CT	Left DLPFC	10 Hz	–	③
Yu Xiaolan, 2022	China	41/41	62.62 ± 5.65/62.83 ± 5.72	3.08 ± 1.41/3.20 ± 1.43	LF-rTMS + CT/CT	Right DLPFC	1 Hz	800	③
Chao, 2020	China	14/14	63.21 ± 7.28/61.57 ± 13.25	6.34 ± 1.50/7.20 ± 1.53	LF-rTMS + CT/CT	Right DLPFC	1 HZ	1000	③
Wenjing et al., 2014	China	30/30	63.73 ± 6.07/65.24 ± 5.42	3.93 ± 4.78/4.21 ± 4.25	LF-rTMS + CT/CT	–	1 Hz	–	③
Qingping et al., 2022	China	50/50	65.07 ± 3.58/64.52 ± 3.67	6.03 ± 1.12/5.96 ± 1.07	HF-rTMS + CT/LF-rTMS + CT	Posterolateral to the left prefrontal lobe	10 Hz/1 Hz	1600/1000	③
Hua et al., 2019	China	20/20	61.40 ± 6.72/60.15 ± 7.44	2.03 ± 0.68/2.05 ± 0.86	HF-rTMS + CT/CT	Left DLPFC	10 Hz	–	③
Zhang and Sha, 2022	China	29/29	64.58 ± 7.28/66.32 ± 5.44	6.84 ± 1.50/7.00 ± 1.53	HF-rTMS + CT/CT	Left DLPFC	5 Hz	1600	③
Shin et al., 2016	South Korea	10/8	69.00 ± 20.00/67.00 ± 18.51	7.00 ± 1.54/4.91 ± 1.46	HF-rTMS + CT/CT + Sham	Left DLPFC	5 Hz	600	③
Jiang et al., 2023	China	29/28	62.7 ± 12.9/64.3 ± 8.9	7.00 ± 5.33/3.00 ± 3.70	HF-rTMS + CT/CT + Sham	Left DLPFC	10 Hz	1200	③
Chen et al., 2022	China	29/29	64.17 ± 8.37/63.70 ± 8.88	4.14 ± 2.47/3.79 ± 3.07	HF-rTMS + CT/CT	Left DLPFC	5 Hz	–	③
Brys et al., 2016	America	14/15	64.6 ± 12.3/64.0 ± 7.4	4.5 ± 2.2/7.7 ± 4.2	HF-rTMS + CT/CT + Sham	Left DLPFC	10 Hz	–	③

E, experimental group; C, control group; HF-rTMS, high-frequency repetitive transcranial magnetic stimulation; LF-rTMS, low-frequency repetitive transcranial magnetic stimulation; CT, Conventional treatment; DLPFC, dorsolateral prefrontal Cortex; SMA, supplementary motor area; ①PSQI, Pittsburgh Sleep Quality Index; ②PDSS, Parkinson’s Disease Sleep Scale; ③HAMD, Hamilton Depression Scale; –, not mentioned.

### 3.3 Quality evaluation

**FIGURE 2:**
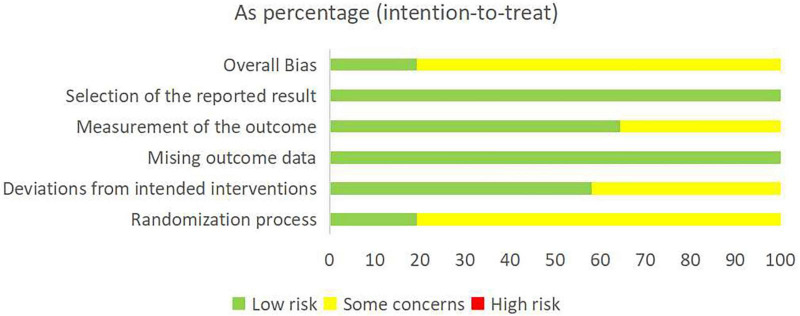
Risk bias assessment plot (green, yellow, and red indicate low, moderate, and high risk levels).

Risk of bias was assessed using the Cochrane Risk of Bias 2.0 tool. Eighteen trials (58.1%) described their randomization process; four (12.9%) reported allocation concealment; eight (25.8%) reported blinding of participants and personnel; and 17 (54.8%) reported blinding of outcome assessment. All research data were complete. Detailed results of the risk-of-bias assessment are provided in Figure 2. Regarding PEDro scores, all studies scored >4 (median 6; range 4–9). See Supplementary Table 2 for details.

### 3.4 Network of evidence

**FIGURE 3:**
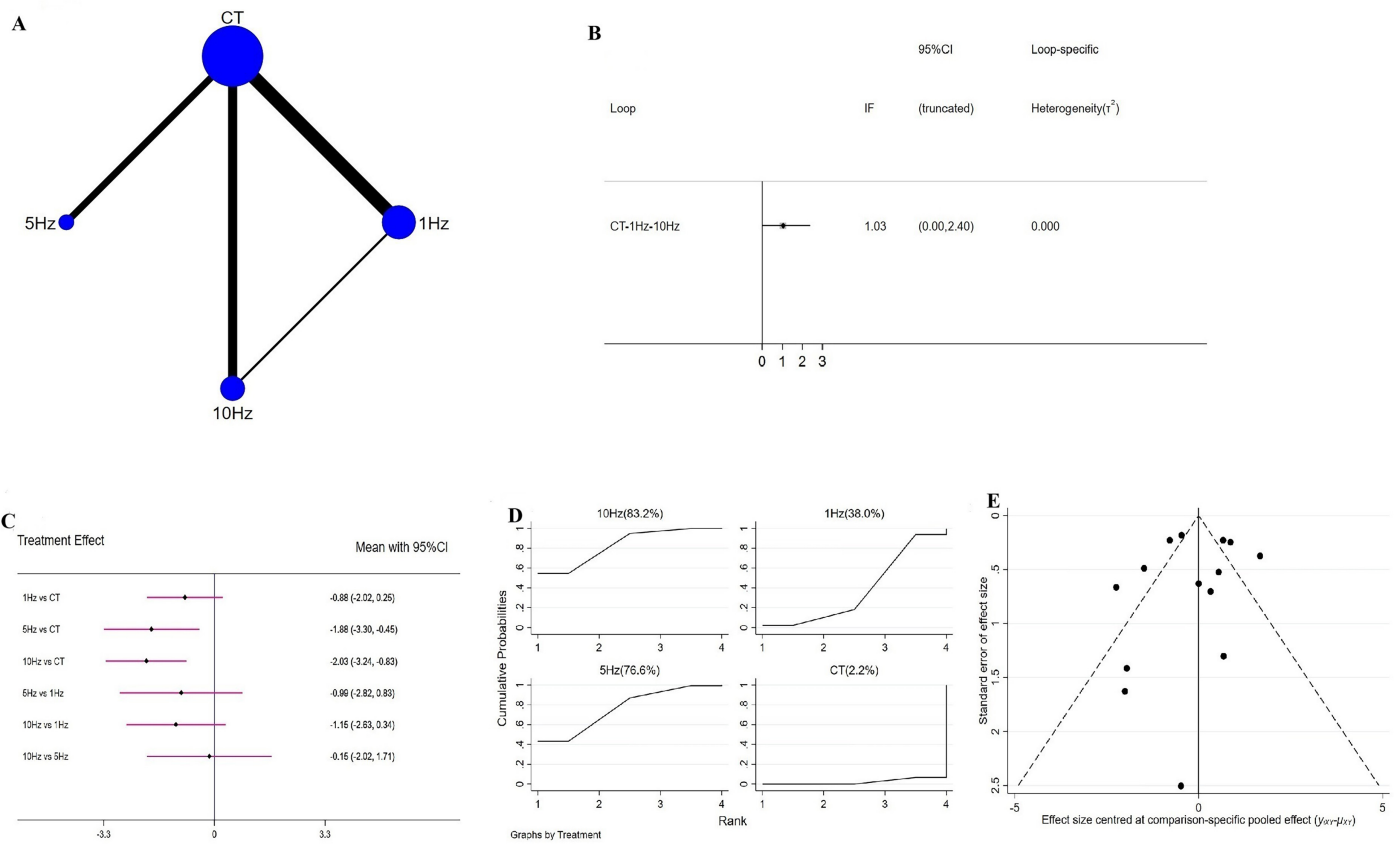
Network meta-analysis results for Pittsburgh Sleep Quality Index (PSQI). **(A)** Network plot; **(B)** ring inconsistencies (*X*-axis, effect size, 0 represents no effect; *Y*-axis, closed loop between interventions, the horizontal line range represents the 95% confidence interval; diamond represents point estimate); **(C)** forest plot (*X*-axis, effect size, with 0 representing no effect; *Y*-axis, comparison between interventions, with the red line range indicating the 95% confidence interval; diamonds representing pooled effect sizes); **(D)** the figure of ranking probability (*X*-axis, ranking position (Rank), *Y*-axis, representing Cumulative Probability); **(E)** funnel plot (*X*-axis, effect size, *Y*-axis, standard error of effect size, and black dots represent the results of each study).

**FIGURE 4:**
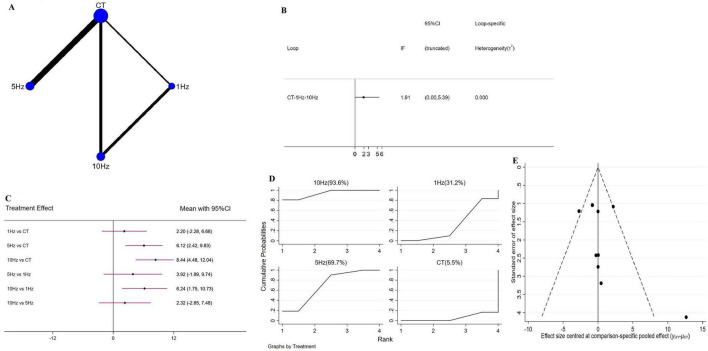
Network meta-analysis results for Parkinson’s Disease Sleep Scale (PDSS). **(A)** Network plot; **(B)** ring inconsistencies (*X*-axis, effect size, 0 represents no effect; *Y*-axis, closed loop between interventions, the horizontal line range represents the 95% confidence interval; diamond represents point estimate); **(C)** forest plot (*X*-axis, effect size, with 0 representing no effect; *Y*-axis, comparison between interventions, with the red line range indicating the 95% confidence interval; diamonds representing pooled effect sizes); **(D)** the figure of Ranking probability (*X*-axis, ranking position (Rank), *Y*-axis, representing Cumulative Probability); **(E)** funnel plot (*X*-axis, effect size, *Y*-axis, standard error of effect size, and black dots represent the results of each study).

**FIGURE 5:**
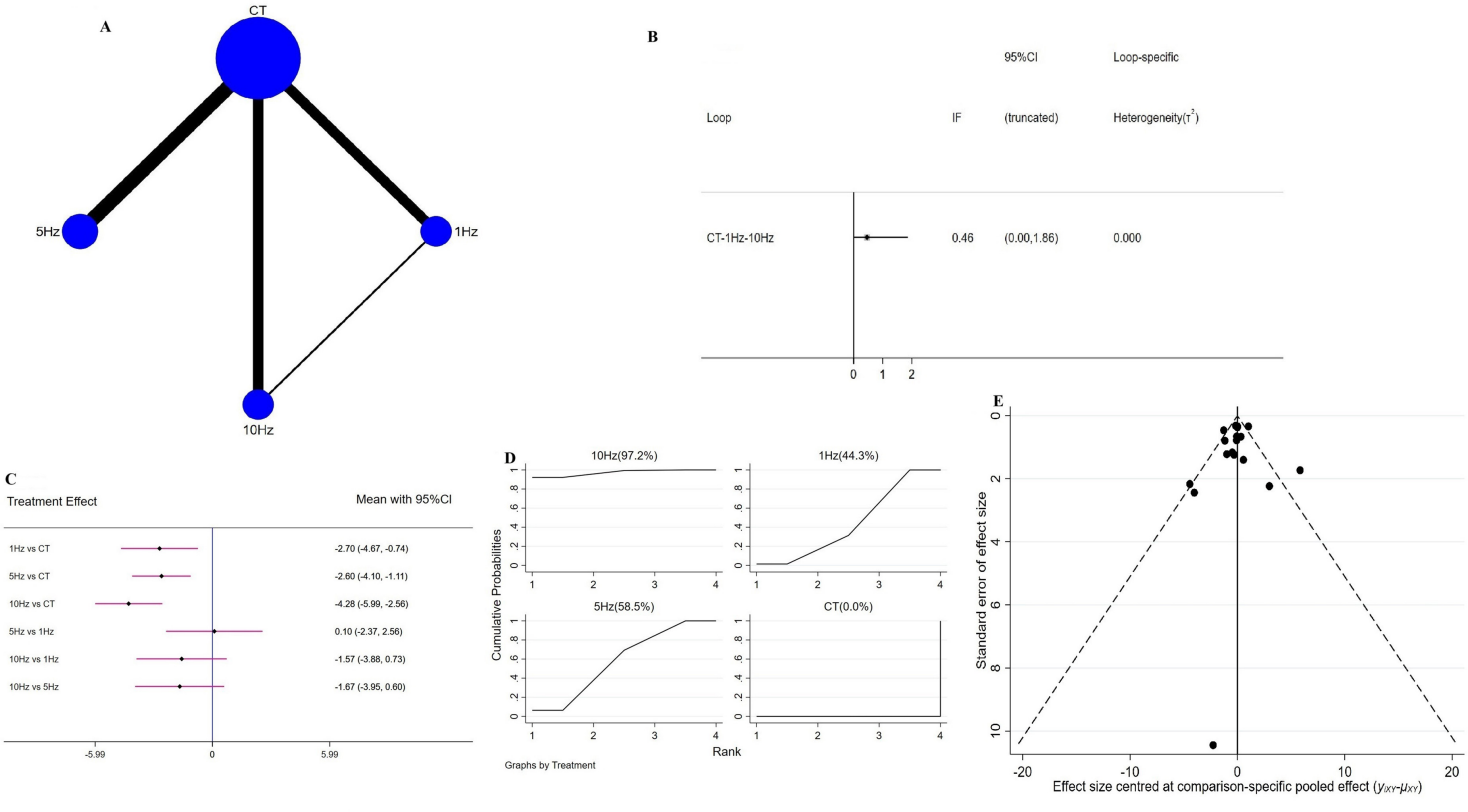
Network meta-analysis results for Hamilton Depression Scale (HAMD). **(A)** Network plot; **(B)** ring inconsistencies (*X*-axis, effect size, 0 represents no effect; *Y*-axis, closed loop between interventions, the horizontal line range represents the 95% confidence interval; diamond represents point estimate); **(C)** forest plot (*X*-axis, effect size, with 0 representing no effect; *Y*-axis, comparison between interventions, with the red line range indicating the 95% confidence interval; diamonds representing pooled effect sizes); **(D)** the figure of Ranking probability (*X*-axis, ranking position (Rank), *Y*-axis, representing Cumulative Probability); **(E)** funnel plot (*X*-axis, effect size, *Y*-axis, standard error of effect size, and black dots represent the results of each study).

Network geometry for each outcome is shown in Figures 3–5. Figure 3A illustrates the four interventions compared on PSQI (with the largest sample in the conventional treatment node); Figure 4A shows the PDSS network of four interventions; and Figure 5A depicts the HAMD network.

#### 3.4.1 rTMS for PSQI

A total of 14 RCTs evaluated the effects of rTMS on PSQI, encompassing four interventions-1 Hz rTMS, 5 Hz rTMS, 10 Hz rTMS, and conventional treatment (CT)-and forming a closed intervention loop. Loop inconsistency testing demonstrated good agreement across studies (IF = 1.03, 95% CI = 0.00 to 2.40) (Figure 3B), permitting use of a consistency model. Compared with CT, both 5 Hz (WMD = −1.88, 95% CI = −3.30 to −0.45) and 10 Hz rTMS (WMD = −2.03, 95% CI = −3.24 to −0.83) significantly improved PSQI, whereas 1 Hz rTMS (WMD = −0.88, 95% CI = −2.02 to 0.25) produced a non-significant reduction (Figure 3C); no pairwise differences among frequencies reached significance. SUCRA ranking indicated that 10 Hz rTMS had the highest probability of being the optimal intervention (SUCRA = 83.2%), followed by 5 Hz (76.6%), 1 Hz (38.0%) and CT (2.2%) (Figure 3D). Sensitivity analysis-excluding studies with sample sizes <10-yielded identical SUCRA orderings, reaffirming 10 Hz rTMS as the most effective. Pairwise comparisons displayed in forest plots likewise mirrored the overall findings (Supplementary Figure 5).

#### 3.4.2 rTMS for PDSS

A total of eight RCTs evaluated the effect of rTMS on PDSS, comprising four interventions, 1 Hz rTMS, 5 Hz rTMS, 10 Hz rTMS, and conventional treatment (CT), which together formed a closed intervention loop. Loop inconsistency testing demonstrated good agreement (IF = 1.91, 95% CI = 0.00 to −5.39) (Figure 4B), permitting analysis via a consistency model. Compared with CT, both 5 Hz (WMD = 6.12, 95% CI = 2.42 to 9.83) and 10 Hz rTMS (WMD = 8.44, 95% CI = 4.84 to 12.04) produced significant improvements in PDSS scores, whereas 1 Hz rTMS (WMD = 2.20, 95% CI = −2.28 to 6.68) yielded a non-significant reduction. In head-to-head comparisons, 10 Hz outperformed 1 Hz (WMD = 6.24, 95% CI = 1.75 to 10.73), with no other pairwise differences reaching significance (Figure 4C). SUCRA ranking indicated that 10 Hz rTMS was the most likely optimal intervention (93.6%), followed by 5 Hz (69.7%), 1 Hz (31.2%), and CT (5.5%) (Figure 4D). No sensitivity analysis was performed for this outcome, as all included studies had sample sizes ≥10.

#### 3.4.3 rTMS for HAMD

A total of 18 RCTs assessed rTMS effects on HAMD, using the same four interventions–1 Hz rTMS, 5 Hz rTMS, 10 Hz rTMS, and CT–which also formed a closed network. Loop inconsistency testing again showed good agreement (IF = 0.46, 95 CI = 0.00 to 1.86) (Figure 5B), justifying use of a consistency model. Versus CT, each rTMS frequency 1 Hz (WMD = −2.70, 95% CI = −4.67 to −0.74), 5 Hz (WMD = −2.60, 95% CI = −4.10 to −1.11) and 10 Hz (WMD = −4.28, 95% CI = −5.99 to −2.56) significantly reduced HAMD scores, with no significant differences in pairwise frequency comparisons (Figure 5C). SUCRA ranking identified 10 Hz as the best intervention (97.2%), followed by 5 Hz (58.5%), 1 Hz (44.3%), and CT (0.0%) (Figure 5D). Sensitivity analysis–excluding studies with sample sizes <10–yielded the same SUCRA order and mirrored the overall forest-plot results (Supplementary Figure 6).

### 3.5 Subgroup

Previous studies have demonstrated that varying the number of rTMS pulses may elicit dose-dependent remodeling of neuronal networks in PD patients (Anil et al., 2023). Accordingly, we stratified analyses into two subgroups based on total pulse count: 600-pulse and >600-pulse.

#### 3.5.1 600 pulse subgroup

In the 600-pulse PSQI subgroup, three RCTs comparing 1 Hz and 5 Hz rTMS were pooled using a consistency model. Relative to conventional treatment (CT), 5 Hz rTMS significantly reduced PSQI scores (WMD = −1.72, 95% CI = −2.18 to −1.27), whereas 1 Hz rTMS was inferior to CT (WMD = 1.12, 95% CI = 0.39 to 1.85), likely reflecting the subgroup’s small sample size. A direct comparison confirmed superior PSQI improvement with 5 Hz versus 1 Hz (WMD = −2.84, 95% CI = −3.71 to −1.98). SUCRA ranking designated 5 Hz as the optimal intervention (SUCRA = 100.0%), followed by CT (49.9%) and 1 Hz (0.1%) (Supplementary Figure 1). Only one RCT has examined 600-pulse rTMS for PDSS, and literature on 600-pulse rTMS effects on HAMD is similarly limited; thus, subgroup analyses for these outcomes were not performed.

#### 3.5.2 Subgroup with >600 pulses

In the >600-pulse PSQI subgroup, ten RCTs comparing 1 Hz and 10 Hz rTMS in PD patients were pooled using a consistency model after demonstrating good network agreement (*p* > 0.05). Both 1 Hz (WMD = −1.72, 95% CI = −2.87 to −0.58) and 10 Hz rTMS (WMD = −2.09, 95% CI = −3.51 to −0.68) yielded significant PSQI improvements versus conventional treatment (CT), with no significant difference observed between the two frequencies (WMD = −0.37, 95% CI = −2.00 to 1.26). SUCRA ranking placed 10 Hz first (83.3%), followed by 1 Hz (66.5%) and CT (0.2%) (Supplementary Figure 2).

In the >600-pulse PDSS subgroup, seven RCTs involving 1 Hz, 5 Hz and 10 Hz rTMS were analyzed under a consistency model (loop inconsistency *p* > 0.05). Both 5 Hz (WMD = 6.12, 95% CI = 1.79 to 10.44) and 10 Hz rTMS (WMD = 9.25, 95% CI = 5.43 to 13.8) significantly enhanced PDSS scores compared to CT, whereas 1 Hz (WMD = 0.55, 95% CI = −4.69 to 5.79) did not. Head-to-head comparisons showed 10 Hz to be superior to 1 Hz (WMD = 8.70, 95% CI = 2.61 to 14.79), with no other pairwise differences reaching significance. SUCRA ranked 10 Hz highest (95.1%), then 5 Hz (69.6%), 1 Hz (21.3%) and CT (14.1%) (Supplementary Figure 3).

In the >600-pulse HAMD subgroup, eleven RCTs assessing 1 Hz, 5 Hz and 10 Hz rTMS formed a closed network with good consistency (*P* > 0.05). All three frequencies significantly reduced HAMD scores versus CT (1 Hz WMD = −2.14, 95% CI = −2.88 to −1.40, 5 Hz WMD = −2.40, 95% CI = −2.95 to −1.85 and 10 Hz WMD = −3.97, 95% CI = −4.54 to −3.40). Pairwise analyses revealed that 10 Hz outperformed both 1 Hz (WMD = −1.83, 95% CI = −2.46 to −1.19) and 5 Hz (WMD = 1.57, 95% CI = −2.37 to −0.77), while the latter two did not differ significantly. SUCRA indicated 10 Hz as the optimal intervention (100.0%), followed by 5 Hz (56.9%), 1 Hz (43.1%) and CT (0.0%) (Supplementary Figure 4).

### 3.6 Publication bias

Publication bias, evaluated via funnel plots for all outcomes, was broadly symmetrical, with only a few studies lying outside the funnel and suggesting minimal bias (Figures 3E–5E).

### 3.7 Sensitivity analyses

After excluding studies with a PEDro score of less than 6, the research results remained unchanged (Supplementary Figures 7–9). There was only one study with an H-Y stage >3, and even after excluding it, the research results remained unchanged (Supplementary Figures 10, 11). This indicates that the results of this study are relatively stable and reliable.

### 3.8 Adverse reactions

Eight studies provided detailed accounts of adverse effects: 13 patients experienced transient headaches that resolved with rest and were able to complete the protocol, and six patients reported transient dizziness, which likewise subsided after resting (Supplementary Table 3).

## 4 Discussion

This study employed a NMA to evaluate and compare the effects of rTMS at various frequencies, combined with conventional therapy, on sleep disorders and depressive symptoms in patients with Parkinson’s disease. Compared with conventional therapy alone, all rTMS frequencies significantly improved PSQI, PDSS, and HAMD scores, with 10 Hz rTMS appearing to be the most effective for both sleep and mood. We then stratified stimulation by pulse count (600 pulses vs. >600 pulses). In the 600-pulse group, which did not include 10 Hz stimulation, 5 Hz rTMS yielded the greatest benefit; in the >600-pulse subgroup, 10 Hz rTMS produced the most pronounced improvements.

This study employed a NMA to assess and compare the effects of different rTMS frequencies, each combined with conventional therapy, on sleep disorders in PD patients. The findings demonstrated that, relative to conventional treatment alone, all rTMS frequencies significantly improved PSQI, PDSS and HAMD scores, with 10 Hz rTMS plus standard therapy emerging as the most effective intervention for each outcome. There are certain differences in the findings regarding the impact on sleep between this study and that of Cristini et al. (2025). Cristini’s research revealed that LF-rTMS can enhance subjective sleep quality in PD patients, yet the evidence for HF-rTMS improving sleep quality is insufficient. This discrepancy may stem from Cristini’s study not precisely categorizing HF-rTMS by frequency, potentially leading to interference from mixing different frequency groups. Additionally, the patients in the HF-rTMS group in that study had relatively mild sleep issues, which could have contributed to a ceiling effect. Regarding depression, a previous meta-analysis (Zhou et al., 2018) indicated that 5 Hz rTMS is most effective in alleviating depressive symptoms. Upon comparison, we found that the studies included in that meta-analysis were self-controlled before-and-after designs, and the number of included studies was limited. Currently, it is believed that abnormal discharges in the subthalamic nucleus (STN) of PD patients are transmitted through the cortical–striatal–thalamic circuit, leading to disruptions in the sleep–wake cycle. Repetitive TMS is one of the most widely applied neurostimulation modalities: high-frequency rTMS (HF-rTMS), defined as stimulation above 1 Hz has been shown to induce long-term excitatory effects (Valero-Cabré et al., 2017), whereas low-frequency rTMS (LF-rTMS), defined as 1 Hz or below, is expected to produce inhibitory effects and elicit long-term depression (Romero et al., 2002).

Low frequency rTMS reduces sleep fragmentation by attenuating abnormal beta oscillations (20–30 Hz) in the thalamus, subthalamic nucleus (STN) and motor cortex via long-term depression (LTD) (Chen et al., 1997). Simultaneous stimulation of the prefrontal cortex (PFC) increases δ-wave (1–4 Hz) power and prolongs slow-wave sleep. LF-rTMS also upregulates striatal dopamine D_2_-receptor expression, enhances dopaminergic signaling, and alleviates Parkinson’s disease-associated REM sleep behavior disorder (RBD) (Ahmed et al., 2012). Moreover, cortical rTMS promotes the release of dopamine and pineal melatonin, increases brain serotonin and norepinephrine levels, and elevates serum GABA–neurotransmitters critical to the sleep–wake cycle–thereby improving sleep quality and reducing daytime somnolence (Strafella et al., 2003; Feng et al., 2019). High-frequency (HF) rTMS activates the dorsolateral prefrontal cortex (DLPFC) and anterior cingulate cortex (ACC) via long-term potentiation (LTP), inhibits the noradrenergic arousal system in the locus coeruleus (LC), and ameliorates excessive daytime sleepiness (Lefaucheur et al., 2020). Prior studies have shown that HF-rTMS over the parietal lobe enhances deep sleep and sleep efficiency while reducing nocturnal awakenings in PD patients, suggesting the parietal cortex as a key target for deepening subsequent sleep by decreasing Stage I and increasing Stage IV sleep (van Dijk et al., 2009). HF-rTMS also augments cortical excitability, improves cerebral blood flow, and promotes endogenous dopamine release, thereby modulating excitation within the direct and indirect striatal–pallidal pathways, which may further alleviate sleep disturbances (Chou et al., 2015; Qin et al., 2018). The superior efficacy of 10 Hz rTMS observed here may reflect dose-dependent neuroplastic changes: higher pulse counts strengthen neural network connectivity and induce sustained synaptic potentiation, enhancing neuromodulatory potential (Zhou et al., 2018; Anil et al., 2023). This dose dependency is supported by our subgroup analysis, which indicates that stimulation dosage differentially affects outcomes in PD patients.

In our network meta-analysis of depressive symptoms, hyporeactivity of the left DLPFC has been implicated in PD-related depression (Mottaghy et al., 2002). Clinical protocols therefore aim to increase left DLPFC excitability while inhibiting right DLPFC activity: LF-rTMS to the right DLPFC reduces cortical excitability, diminishing negative affect and trans-synaptically activating the hypoactive left DLPFC (Grimm et al., 2008). Conversely, HF-rTMS elicits release of dopamine, serotonin (5-HT), glutamate, and brain-derived neurotrophic factor (BDNF). Because depression in PD involves deficits in dopaminergic and serotonergic systems, rTMS may improve mood through dual-transmitter regulation (Strafella et al., 2001). HF-rTMS targeting the DLPFC also modulates prefrontal–limbic functional connectivity (e.g., amygdala, ACC) by enhancing local neuronal excitability, inhibiting aberrant default mode network (DMN) activity, and strengthening frontal regulation of limbic regions (Lefaucheur et al., 2020). Furthermore, the observed correlation between sleep quality and mood, namely, PD patients with poor sleep exhibit more severe depression than those with normal sleep, suggests that amelioration of sleep disturbances may contribute to improvements in depressive symptoms. Patients with neurodegenerative diseases often exhibit higher rates of depression than the general population (Tandberg et al., 1998). Dopaminergic dysfunction is hypothesized to underlie the strong association between poor sleep quality and depression severity in Parkinson’s disease (PD). In healthy individuals, sleep deprivation elicits a compensatory increase in central dopamine levels; however, PD-related dopamine deficits may impair this adaptive response, thereby exacerbating depressive symptoms (Kay et al., 2018). Moreover, improvements in HAMD anxiety scores have been positively correlated with PSQI improvements, suggesting that enhanced sleep quality is associated with reduced anxiety (Huang et al., 2018).

The use of dopaminergic drugs may also affect the efficacy of rTMS. Previous studies (Fierro et al., 2008) found that 10 Hz rTMS only enhances cortical inhibition during drug withdrawal in PD patients, whereas the improvement in cortical inhibition during medication use is comparable to that of the drugs themselves. All subjects included in this study were on dopaminergic drugs during the trial period. The reason for the divergence may be related to the large number of subjects included in this study–all of whom were randomized controlled trials–as well as differences in intervention methods and targets. Fierro et al. (2008) used 10 Hz, 500-pulse stimulation over the M1 region, while most studies in this review used 10 Hz, 1200-pulse stimulation, with the stimulation targets mostly being the DLPFC, which may also account for the differences in results. The stage of PD is another factor affecting the efficacy of rTMS. Flamez et al. (2016) found that LF-rTMS did not significantly improve motor function in PD patients, possibly because all subjects in their study were late-stage PD patients (H&Y ≥ 3). In this study, most subjects in the included literature were in H&Y stages 1–3 (only one was a late-stage patient; after sensitivity analysis, the results remained unchanged; see Supplementary Figures 10, 11), which also explains the differences between this study’s results and those of previous studies. Meanwhile, previous studies (Cong et al., 2022) have also shown that the degree of sleep disturbance and depression in PD patients is positively correlated with H&Y stage. Late-stage PD patients have extensive neurodegenerative lesions, and local stimulation may not be able to regulate distant pathological networks.

Regarding adverse events reported in this study, only a small number of subjects experienced transient dizziness, headache, or scalp numbness, which resolved after rest and allowed them to complete the trial. This indicates the safety of rTMS treatment for PD patients and supports its clinical application. The funnel plot results showed overall symmetry but with a small number of scatter points outside the funnel, so the findings should be interpreted with caution, and more high-quality studies are needed for future verification.

## 5 Limitation

Several limitations should be acknowledged. First, rTMS target regions and pulse counts varied across the included studies, limiting the generalizability of our findings. Second, previous studies (Chung et al., 2019) have indicated that high-level estrogen exposure during HF-rTMS stimulation can enhance the neuroplasticity effect of the prefrontal cortex, suggesting that gender may also influence stimulation outcomes. This study includes a mixed-gender sample from the literature, making subgroup analysis impossible. Third, the severity of sleep disturbance correlates positively with age in PD, yet all participants in the analyzed studies were over 60 years old, precluding age-stratified subgroup analyses. Therefore, future research can focus on more personalized designs for rTMS stimulation targets, pulse counts, gender, and age to provide references for clinical applications.

## 6 Conclusion

In summary, this analysis demonstrates the potential of different rTMS frequencies to ameliorate sleep disturbances and depressive symptoms in PD patients. Notably, 10 Hz rTMS emerged as the most effective intervention for both outcomes. These results provide clinicians and researchers with valuable guidance for managing non-motor symptoms in PD.

## Data Availability

The original contributions presented in this study are included in this article/Supplementary material, further inquiries can be directed to the corresponding author.
