# Supplementary material for: Effects of different frequencies of repetitive transcranial magnetic stimulation on sleep disorders and depression in patients with Parkinson’s disease: a systematic review and network meta-analysis
Source: Front Aging Neurosci. 2025 Sep 8;17:1623917. doi: 10.3389/fnagi.2025.1623917 (PMC12451044; doi:10.3389/fnagi.2025.1623917)
Supplement: Supplementary file 1 [file Data_Sheet_1.pdf]

Supplementary Material

1 Supplementary Data

1. Supplementary Tables

Supplementary Table 1 Search Strategy (using PubMed as an example)

**PubMed**	
**Search Number**	**Query**
#1	“Parkinson Disease”[Mesh]
#2	((((Parkinson[Title/Abstract])OR (Parkinson's[Title/Abstract])) OR(parkinson s disease[Title/Abstract])) OR (parkinsonism[Title/Abstract])) OR (parkinsonian"[Title/Abstract])
#3	#1OR #2
#4	(((repetitive transcranial magnetic stimulation[Title/Abstract]) OR (Transcranial Magnetic Stimulation[Title/Abstract])) OR (TMS[Title/Abstract])) OR (rTMS[Title/Abstract])
#5	#3 AND #4
#6	((((Sleep[Title/Abstract]) OR (sleep disorders[Title/Abstract])) OR (sleep quality[Title/Abstract])) OR (depression[Title/Abstract])) OR (non motor symptoms[Title/Abstract])
#7	#5 AND #6

Supplementary Table 2 Methodological quality of included studies

Reference	Random allocation (0/1)	Concealed allocation (0/1)	Comparableat baseline (0/1)	Blinded subjects (0/1)	Blind therapists(0/1)	Blinded assessors (0/1)	follow-up (0/1)	Intention to-treat analysis (0/1)	Between group comparisons (0/1)	Point estimates and variability (0/1)	Summary
(Bai rui, 2021)	0	0	1	0	0	1	1	1	1	1	6
(Ding Ling and Chen Xu, 2018)	1	0	1	0	0	1	1	1	1	1	7
(Hu Xiaoying et al., 2023)	1	0	1	0	0	0	1	0	1	1	5
(Lai Jinghui et al., 2020)	1	0	1	0	0	0	1	1	1	1	6
(Lei Meng and Jicha	0	0	1	0	0	0	1	1	1	1	5

o, 2024)											
(Xue et al., 2023)	1	0	1	0	0	1	1	1	1	1	7
(Ouy ang Gui-lan, 2022)	1	0	1	0	0	1	1	1	1	1	7
(Qin Xi-xiang et al., 2024)	1	0	1	0	0	0	1	1	1	1	6
(Wa ng Yaju n, 2021)	1	0	1	0	0	0	1	1	1	1	6
(jia-jin, 2021)	1	1	1	1	1	1	1	0	1	1	9
(Zhu ohua	1	0	1	0	0	0	1	1	1	1	6

et al., 2013)											
(Yu Wen-wen and Hai-rong, 2017)	1	0	1	0	0	0	1	1	1	1	6
(Fengju et al., 2017)	0	0	1	0	0	0	1	1	1	1	5
(Zhao Rong, 2023)	0	0	1	0	0	0	1	1	1	1	5
(Wu et al., 2024)	1	1	1	1	1	1	1	0	1	1	9
(Zhuang et al., 2020)	1	0	1	1	0	1	1	0	1	1	7

(Li, 2023)	0	0	1	0	0	0	1	1	1	1	5
(Shaheen et al., 2023)	1	0	1	0	0	1	1	1	1	1	7
(Zhang Xiang et al., 2025)	1	1	1	0	1	1	1	0	1	1	8
(Dai Wei-zheng et al., 2021)	0	0	1	0	0	0	1	0	1	1	4
(Wang Dong and Yuanyu, 2021)	1	0	1	0	0	0	1	0	1	1	5
(Yu Xiaol	1	0	1	0	0	0	1	0	1	1	5

an, 2022)											
(chao, 2020)	1	0	1	0	0	1	1	0	1	1	6
(Wenjing et al., 2014)	1	0	1	0	0	1	1	0	1	1	6
(Qingping et al., 2022)	1	0	1	0	0	0	1	0	1	1	5
(Hua et al., 2019)	0	0	1	0	0	0	1	0	1	1	4
(Zhang Kejun and Liu Sha, 2022)	1	0	1	0	0	0	1	1	1	1	6

(Shin et al., 2016)	1	0	1	1	0	0	1	0	1	1	6
(Jiang et al., 2023)	1	0	1	1	1	0	1	0	1	1	7
(Chen et al., 2022)	1	1	1	0	0	0	1	0	1	1	6
(Brys et al., 2016)	1	0	1	1	0	1	1	1	1	1	8

Supplementary Table 3 Adverse effects

Author	Adverse effects
(Dai Wei-zheng et al., 2021)	Fourpatients experienced transient headaches and dizziness, which were relieved after rest
(Ding Ling and Chen Xu, 2018)	One patient experienced dizziness, which was relieved after rest
(Lai Jinghui et al., 2020)	One patient complained of mild scalp numbness and pain after the first stimulation, which did not recur after rest

(Zhuohua et al., 2013)	Two patients experienced transient headaches, which disappeared after rest
(Yu Xiaolan, 2022)	Two patients experienced headaches, which disappeared after rest
(Qingping et al., 2022)	Two patients experienced headaches, which disappeared after rest
(Zhang Kejun and Liu Sha, 2022)	Two patients experienced transient headaches, which disappeared after rest
(Zhang Xiang et al., 2025)	One patient experienced dizziness, which was relieved after rest

2 Supplementary Figures

2.1 Supplementary Figures

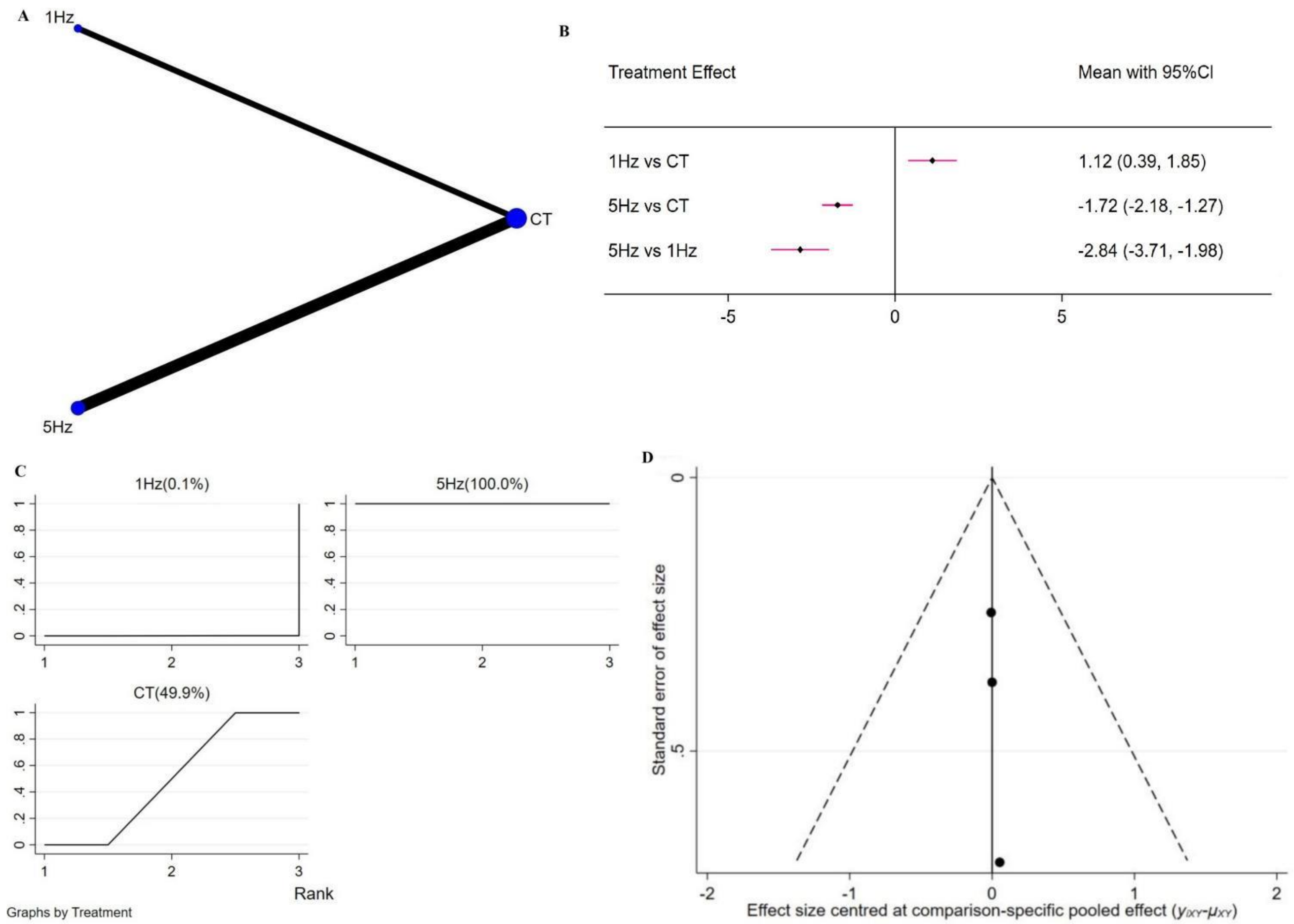

Supplementary Figure 1. Network meta-analysis diagram of PSQI 600 pulse stimulation subgroup analysis. (A): Network plot; (B): Forest plot;(C):The figure ofRankingprobability; (D): :Funnelplot

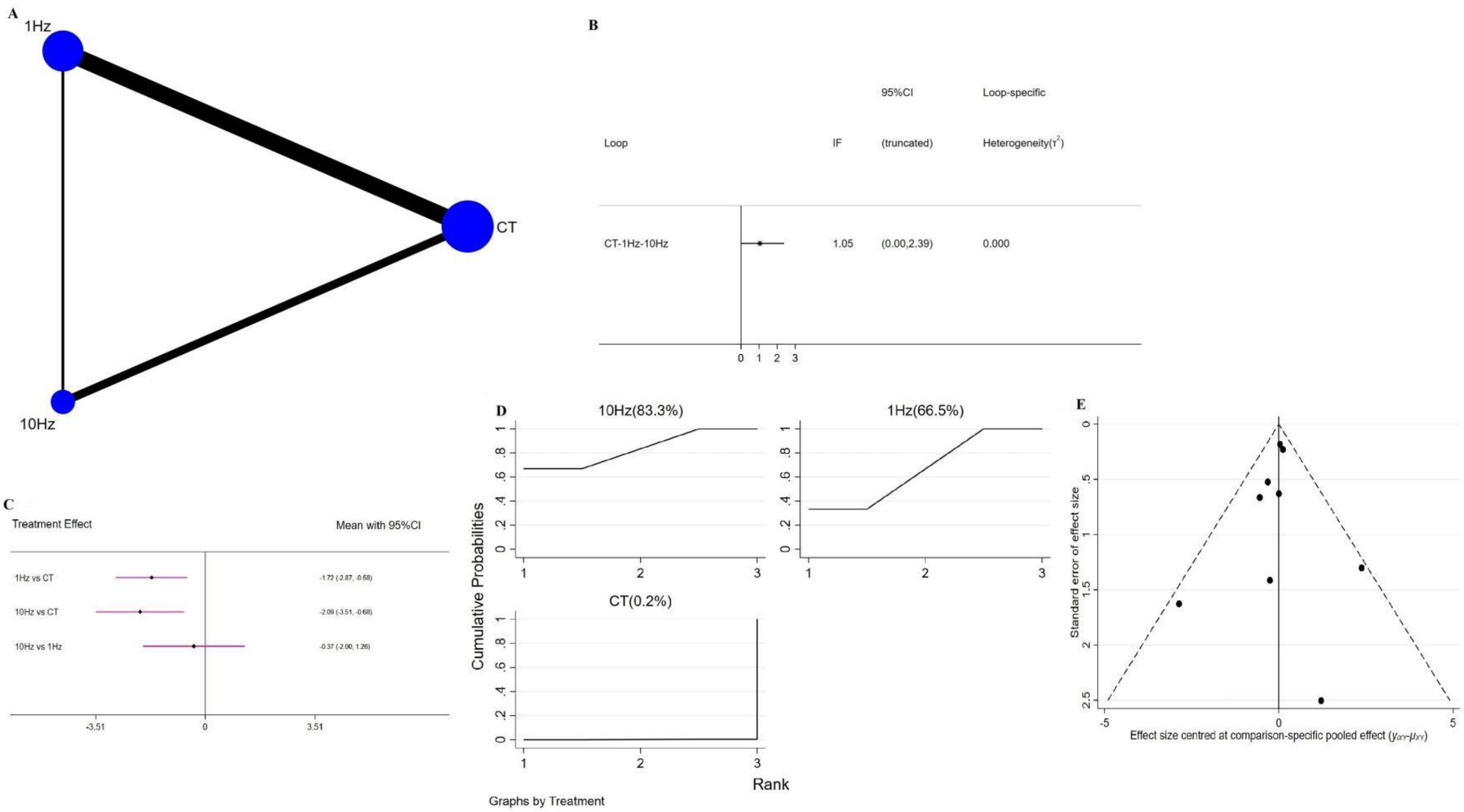

Supplementary Figure 2. Network meta-analysis diagram of PSQI > 600 pulse stimulation subgroup analysis. (A): Network plot; (B):Ring inconsistencies ; (C): Forest plot; (D): The figure of Ranking probability;(E):Funnel plot

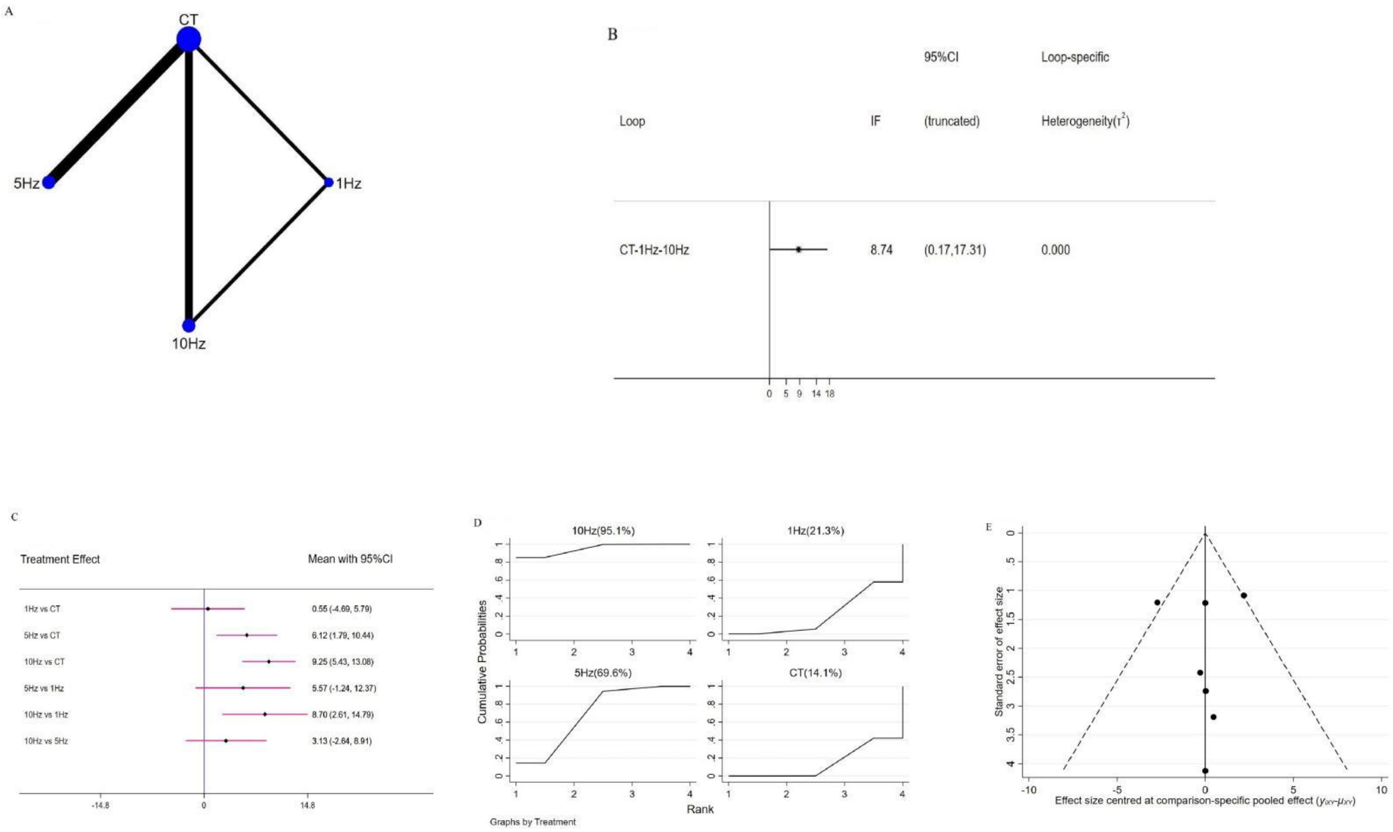

Supplementary Figure 3. Network meta-analysis diagram of PDSS> 600 pulse stimulation subgroup analysis. (A): Network plot; (B):Ring inconsistencies ; (C): Forest plot; (D): The figure ofRankingprobability;(E):Funnelplot

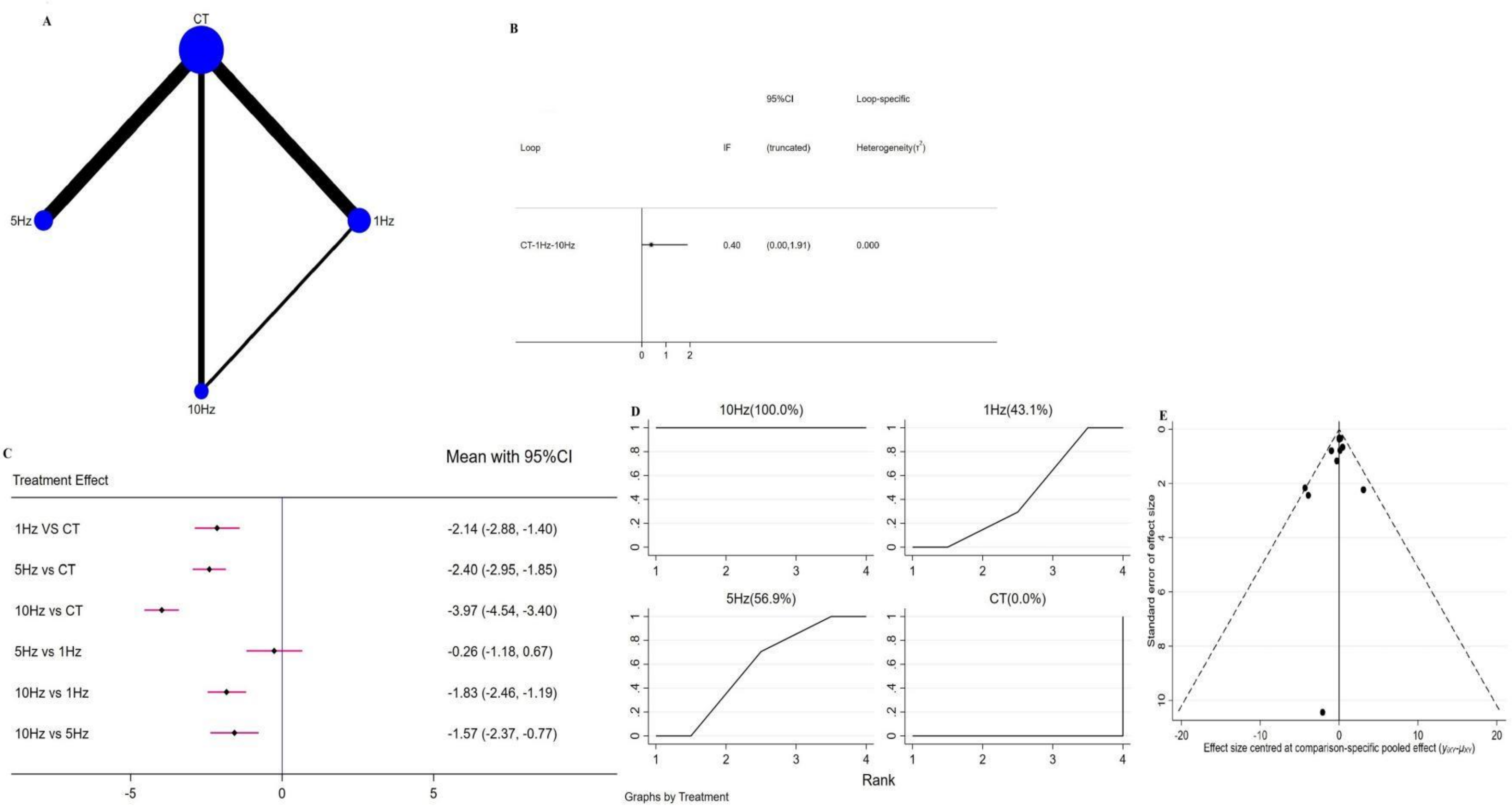

Supplementary Figure 4. Network meta-analysis diagram of HAMD> 600 pulse stimulation subgroup analysis. (A): Network plot; (B):Ring inconsistencies ; (C): Forest plot; (D): The figure ofRankingprobability;(E):Funnelplot

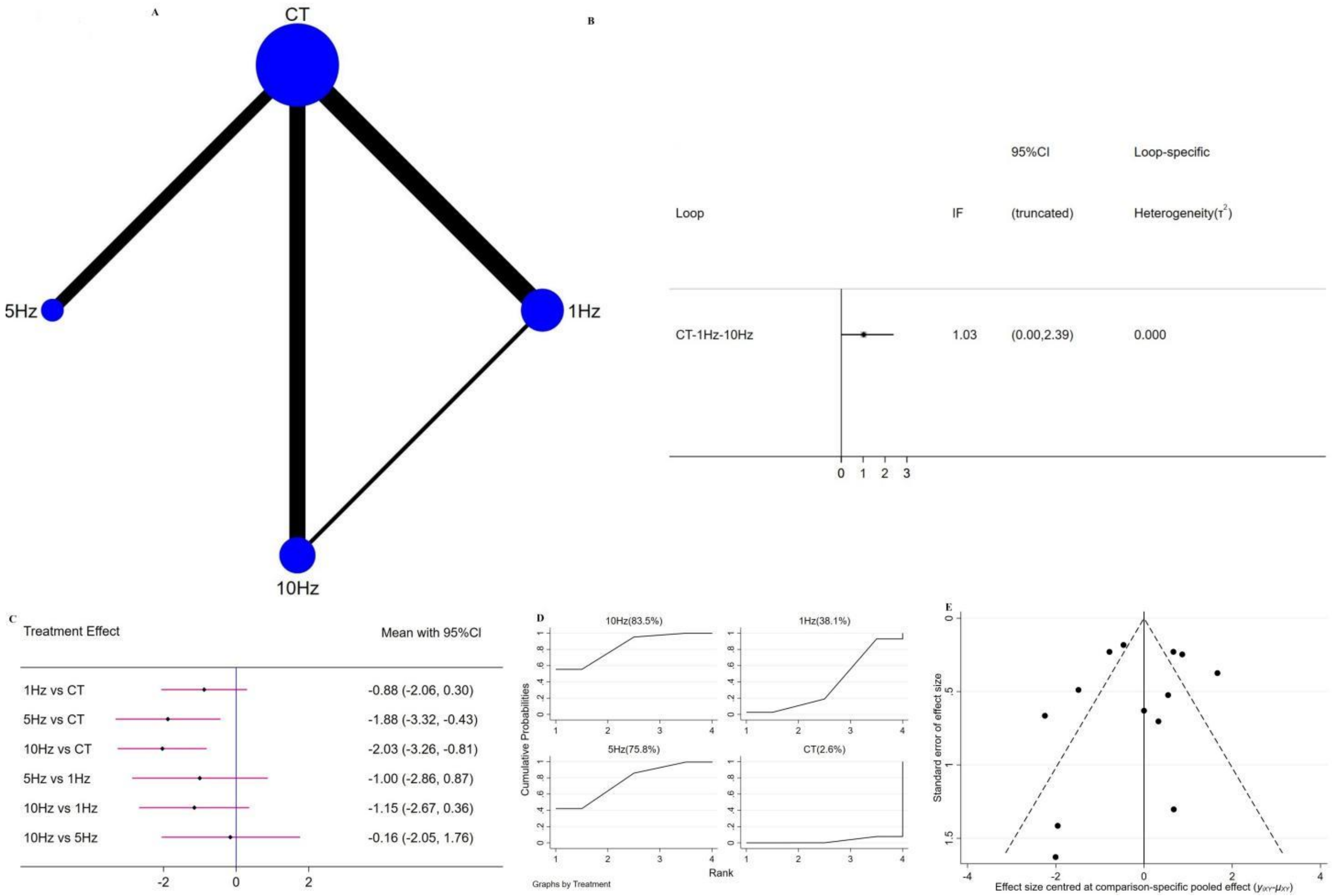

Supplementary Figure 5. Network meta-analysis diagram of PSQI sensitivity analysis(eliminate sample size<10). (A): Network plot; (B):Ring inconsistencies ; (C): Forest plot; (D): The figure ofRankingprobability;(E):Funnelplot

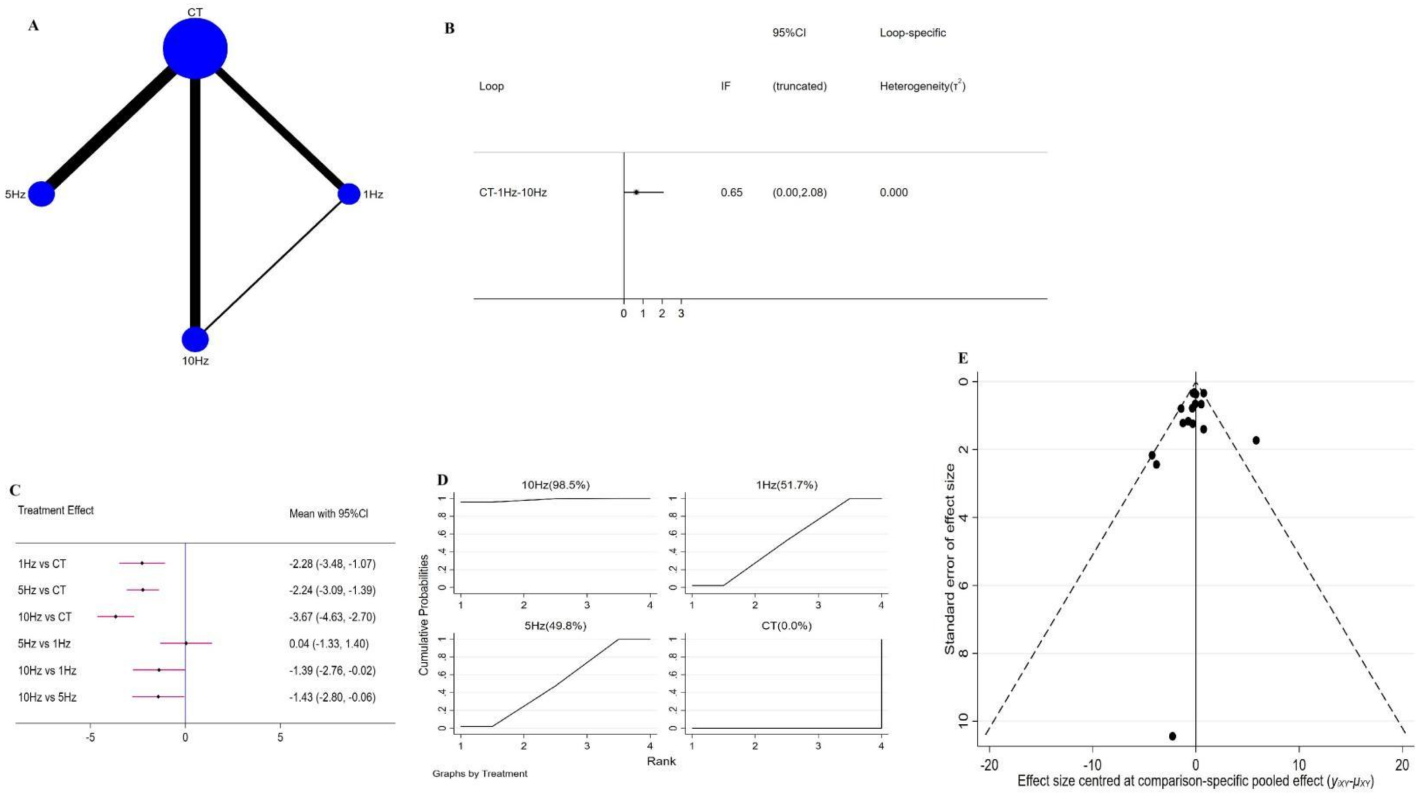

Supplementary Figure 6. Network meta-analysis diagram of HAMD sensitivity analysis(eliminate sample size<10). (A): Network plot; (B):Ring inconsistencies ; (C): Forest plot; (D): The figure ofRankingprobability;(E):Funnelplot

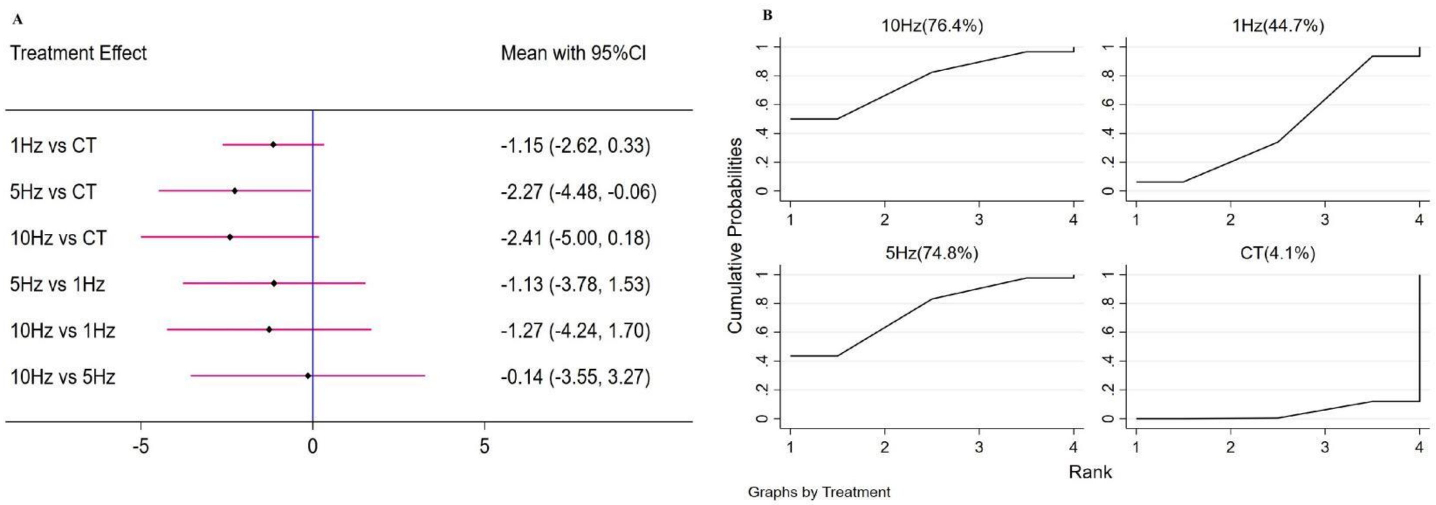

Supplementary Figure 7 Network meta-analysis diagram of PSQI sensitivity analysis(eliminate PEDro<6). (A): Forest plot;(B):The figure ofRankingprobability

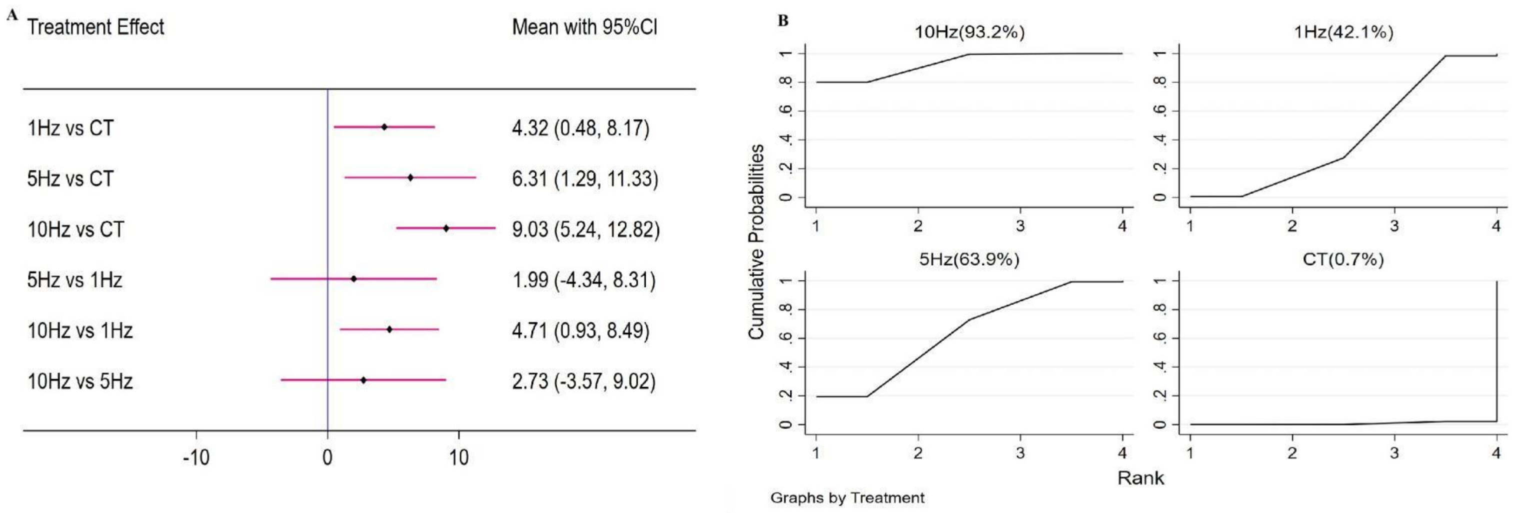

Supplementary Figure 8 Network meta-analysis diagram of PDSS sensitivity analysis(eliminate PEDro<6). (A): Forest plot;(B):The figure ofRankingprobability

Supplementary Figure 9 Network meta-analysis diagram of HAMD sensitivity analysis(eliminate PEDro<6). (A): Forest plot;(B):The figure ofRankingprobability

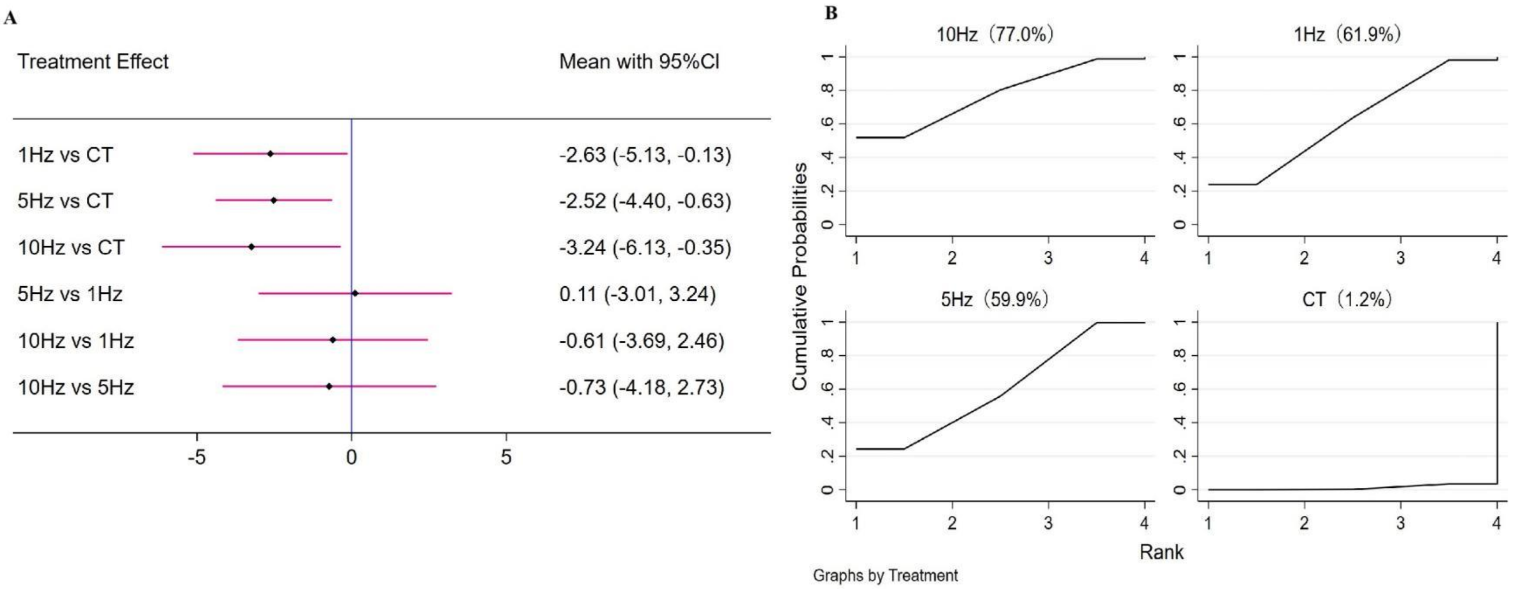

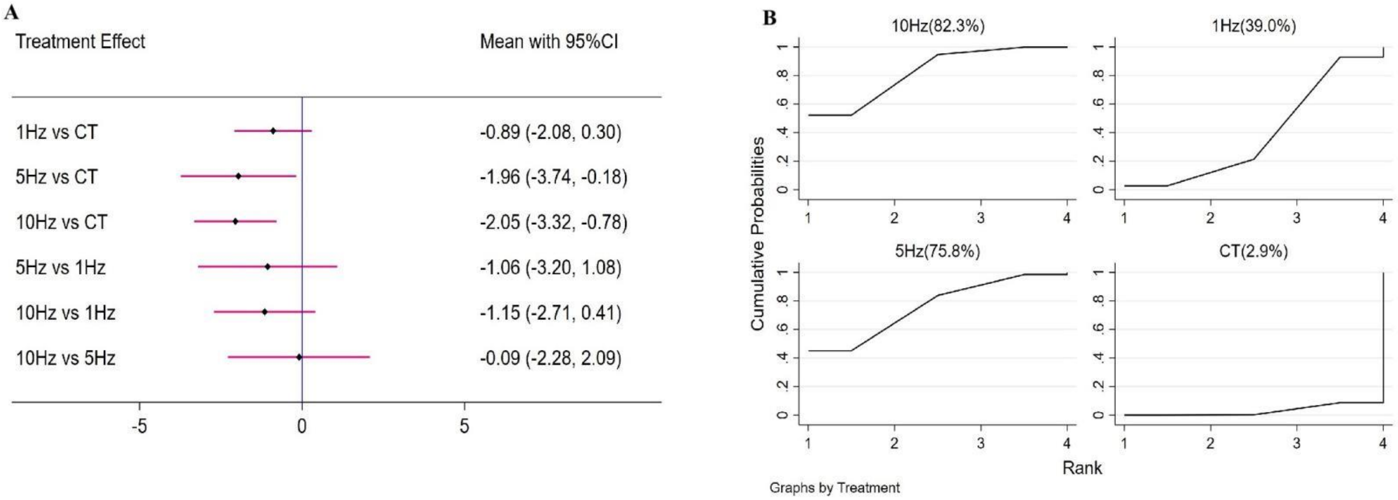

Supplementary Figure 10 Network meta-analysis diagram of PSQI sensitivity analysis(eliminate H-Y>3). (A): Forest plot;(B):The figure ofRankingprobability

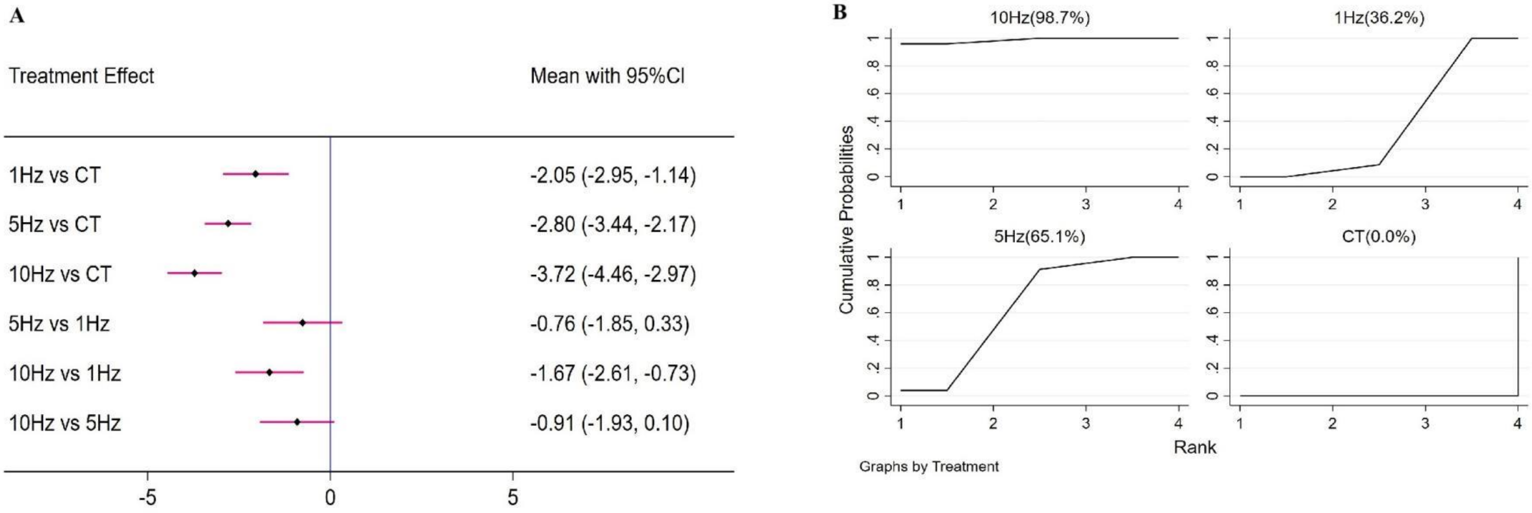

Supplementary Figure 11 Network meta-analysis diagram of HAMD sensitivity analysis(eliminate H-Y>3). (A): Forest plot;(B):The figure ofRankingprobability

REFERENCES

Bai rui, Z. M. Y. X. (2021). Effect of high frequency repetitive transcranial magnetic stimulation in left dorsolateral prefrontal cortex onpatients with Parkinson ’ s disease. *Chinese Journal of Practical Nervous Diseases* 24, 686-691. doi: 10.12083/SYSJ.2021.14.004

Brys, M., Fox, M. D, Agarwal, S., Biagioni, M., Dacpano, G., Kumar, P., et al. (2016). Multifocal repetitive TMS for motor and mood symptoms of Parkinson disease: A randomized trial. *Neurology* 87, 1907-1915. doi: 10.1212/WNL.0000000000003279

chao, Z(2020). Clinical study on low-frequency repetitive transcranial magnetic stimulation for the treatment of Parkinson's disease .Suzhou University

Chen, J, Xu, P, Guo, X, and Zou, T. (2022). Comparative Analysis of the Effects of Escitalopram, Pramipexole, and Transcranial Magnetic Stimulation on Depression in Patients With Parkinson Disease: An Open-Label Randomized Controlled Trial. *Clin. Neuropharmacol.* 45, 84-88. doi: 10.1097/WNF.0000000000000507

Dai Wei-zheng, Fu Mao-lin, and He Wen-qin. (2021). Observation on clinical effects of pramipexole combined with high-frequency repetitive transcranial magnetic stimulation (rTMS) in the treatment of patients suffered from early Parkinson's disease (PD)and depression. *Military Medical Journal of Southeast China* 23, 592-595. doi: 10.3969/j.issn.1672-271X.2021.06.007

Ding Ling, and Chen Xu, H. F. (2018). Effect of Repetitive Transcranial Magnetic Stimulation on Plasma Glu and Gaba Levels in Patients with Parkinson's Sleep Disorder. *China &Foreign Medical Treatment* 37, 7-9,22. doi: 10.16662/j.cnki.1674-0742.2018.26.007

Fengju, Xiaoxue, W., and Xinxin, L. (2017). The therapeutic effect of repetitive transcranial magnetic stimulation combined with cognitive-behavioral therapy on non motor symptoms of Parkinson's disease. *Chinese Journal of Practical Nervous Diseases* 20, 61-63. doi: 10.3969/j.issn.1673-5110.2017.09.024

Hu Xiaoying, Zhang Tong, and Ruiping, W. (2023). Clinical observation of bilateral dorsolateral prefrontal rTMS stimulation in patients with Parkinson's disease. *Chinese Journal of Practical Nervous Diseases* 26, 443-447. doi: 10.12083/SYSJ.221518

Hua, S, Zeshuai, W, Fang, Z, and Xinzhou, J. (2019). A research of repetitive transcranial magnetic stimulation and escitalopram in the treatment of patients with Par-kinson's disease and depression. *Chinese Journal of Practical Nervous Diseases* 22, 1819-1825. doi: 10.12083/SYSJ.2019.16.305

jia-jin, W(2021). The effect of low-frequency repetitive transcranial magnetic stimulation on the clinical symptoms and sleep structure of Parkinson's disease in the dorsolateral prefrontal cortex of the right side .Suzhou University

Jiang, S., Zhan, C., He, P., Feng, S., Gao, Y., Zhao, J., et al. (2023). Neuronavigated repetitive transcranial magnetic stimulation improves depression, anxiety and motor symptoms in Parkinson's disease. *Heliyon* 9, e18364. doi: 10.1016/j.heliyon.2023.e18364

Lai Jinghui, C. Y., Xia Min, Q. L., Wen Jing, Y. L., and Ｒenxiong, Z. (2020). Effects of High-Frequency Ｒtms on Limb Movement and Sleep in Patients with Parkinson's Disease. *World Journal of Sleep Medicine* 7, 1861-1863. doi: 10.3969/j.issn.2095-7130.2020.11.001

Lei Meng, and Jichao, G. (2024). Impact of High -Frequency Repetitive Transcr anial Magnetic Stimulation Combined with Drug Therapy on AutonomicNervous Function, Cognitive Function and Sleep Quality in Patients with Parkinson's Disease. *Clinical Medical & Engineering* 31, 1029-1030. doi: 10.3969/j.issn.1674-4659.2024.09.1029

Li, X. (2023). Application of repetitive transcranial magnetic stimulation with different frequencies in the treatment of Parkinson's sleep disorder. *Med. Sci.*45(3)232-235.doi: 10.3760/cma.j.issn.0254-1424.2023.03.008

Ouyang Gui-lan, (2022). Clinical study of repetitive transcranial magnetic stimulation in the treatment of neuropathic pain associated with Parkinson's disease. *JOURNAL OF GANNAN MEDICAL UNIVERSITY* 42, 805-808. doi: 10.3969/j.issn.1001-5779.2022.08.004

Qin Xi-xiang, Mai Yong-jia, and Zi-qing, (2024). Clinical efficacy of repetitive transcranial magnetic stimulation and pramipexole in mid and late Parkinson’s disease with depression. *JOURNAL OF GUANGDONG MEDICAL UNIVERSITY* 42, 186-189. doi: 10.3969/j.issn.1005-4057.2024.02.013

Qingping, S, Lianhong, H, Wei, X, Shuying, G, Cute, C, Enyu, C, et al. (2022). Comparison of Clinical Effects of Low Frequency and High Frequency Repetitive Transcranial Magnetic Stimulation in the Treatment of Patients with Parkinson Disease. *Chinese and Foreign Medical Research* 20, 14-17. doi: 10.14033/j.cnki.cfmr.2022.22.004

Shaheen, H. A, Gomaa, M, Maarouf, and Daker, (2023). Exploring the effect of transcranial magnetic stimulation on quality of sleep in Parkinson’s disease. *Egypt J Neurol Psychiatr Neurosurg* 59, 173. doi: 10.1186/s41983-023-00771-y

Shin, H. W, Youn, Y. C, Chung, S.J, and Sohn, Y H. (2016). Effect of high-frequency repetitive transcranial magnetic stimulation on major depressive disorder in patients with Parkinson's disease. *J. Neurol.* 263, 1442-1448. doi: 10.1007/s00415-016-8160-x

Wang Dong, and Yuanyu, R. (2021). Clinical efficacy of high-frequency repetitive transcranial magnetic stimulation in patients with Parkinson's disease complicated with depression. *Journal of International Psychiatry* 48, 65-69.

Wang Yajun, (2021). Effect of pramipexole combined with rtms on sleep disturbance in patients with parkinson's disease. *Journal of Shandong Second Medical University* 43, 172-174. doi: 10.16846/j.issn.1004-3101.2021.03.004

Wenjing, Z., Kun, N, Yuhu, Z, Huigen, H, Shaofang, L, and Ruiping, G. (2014). Observation of the Effect of Repetitive Transcranial Magnetic Stimulation in Treating Insomnia Patients with Parkinson's Disease. *Journal of Nursing* 21, 28-30. doi: CNKI:SUN:NFHL.0.2014-23-010

Wu, J, Zhuang, S, Zhang, X, Wang, L, Ma, X, Jin, H, et al. (2024). Objective sleep enhancement in Parkinson's disease: A sham-controlled trial of low-frequency repetitive transcranial magnetic stimulation over the right dorsolateral prefrontal cortex. *Parkinsonism Relat. Disord.* 126, 107050. doi: 10.1016/j.parkreldis.2024.107050

Xue, L, Siyuan, C, Shaopu, W, Qi, G, Dongsheng, L, Weiwei, et al. (2023). The effect of repetitive transcranial magnetic stimulation on sleep and plasma orexin-A levels in patients with advanced Parkinson's disease. *Chin J Phys Med Rehabil* 45, 232-235. doi: 10.3760/cma.j.issn.0254-1424.2023.03.008

Yu Wen-wen, and Hai-rong, S. (2017). Clinical investigation of repetitive transcranial magnetic stimulation on treating depression and sleep disorder in patients with Parkinson's disease in early stage. *J. Clin. Neurol.* 30, 341-345. doi: 10.3969/j.issn.1004-1648.2017.05.007

Yu Xiaolan, C. H. (2022). Clinical Effect and Prognosis Analysis of Repeated Transcranial Mag-netic Stimulation in the Treatment of Patients with Parkinson's Disease and Insomnia. *China &Foreign Medical Treatment* 41, 20-24. doi: 10.16662/j.cnki.1674-0742.2022.04.020

Zhang Kejun, and Liu Sha. (2022). Effectiveness of high-frequency repetitive transcranial magnetic stimulation on the depression, anxiety and quality of life of Parkinson disease patients. *Acta Academiae Medicinae Xuzhou* 42, 885-888. doi: 10.3969/j.issn.2096-3882.2022.12.005

Zhang Xiang, Chen Jing, and Xueling, Z. (2025). Clinical Efficacy of Repetitive Transcranial Magnetic Stimulation Combined with Entacapone-Levodopa-Carbidopa on Motor and Non-motor Symptoms in Parkinson's Disease:a Randomized Controlled Trial. *Chinese General Practice* 28, 581-586,593. doi: 10.12114/j.issn.1007-9572.2024.0411

Zhao Rong, Y. C. (2023). Clinical efficacy of high-low frequency interactive rTMS in the treatment of mid-stage Parkinson's disease. *Journal of Yan'an University(Medical Science Edition)* 21, 59-63,68. doi: 10.19893/j.cnki.ydyxb.2023-0076

Zhuang, S, Wang, Gu, X, Wu, Mao, Gui, H, et al. (2020). Low-Frequency Repetitive Transcranial Magnetic Stimulation over Right Dorsolateral Prefrontal Cortex in Parkinson's Disease. *Parkinson's disease* 2020, 7295414. doi: 10.1155/2020/7295414

Zhuohua, W, Liqian, C, Ming, S, Hongyu, T, Yijuan, W, Qifeng, X, et al. (2013). Clinical Observation of Repetitive Transcranial Magnetic Stimulation for Treating Insomnia in Parkinson's Disease. *Journal of Jiangsu University(Medicine Edition)* 23, 354-356. doi: CNKI:SUN:ZJYZ.0.2013-04-020
